# Machine Learning Unmasked Nutritional Imbalances on the Medicinal Plant *Bryophyllum* sp. Cultured *in vitro*

**DOI:** 10.3389/fpls.2020.576177

**Published:** 2020-12-01

**Authors:** Pascual García-Pérez, Eva Lozano-Milo, Mariana Landin, Pedro Pablo Gallego

**Affiliations:** ^1^Applied Plant and Soil Biology, Plant Biology and Soil Science Department, Biology Faculty, University of Vigo, Vigo, Spain; ^2^Clúster de Investigación e Transferencia Agroalimentaria do Campus da Auga - Agri-Food Research and Transfer Cluster, University of Vigo, Ourense, Spain; ^3^Grupo I+D Farma (GI-1645), AeMat, Pharmacology, Pharmacy and Pharmaceutical Technology Department, Pharmacy Faculty, University of Santiago, Santiago de Compostela, Spain; ^4^Health Research Institute of Santiago de Compostela (IDIS), Santiago de Compostela, Spain

**Keywords:** *Kalanchoe*, ANNs, media mineral nutrition, plant *in vitro* culture, artificial intelligence algorithms, structural risk minimization (SRM), ASMOD

## Abstract

Plant nutrition is a crucial factor that is usually underestimated when designing plant *in vitro* culture protocols of unexploited plants. As a complex multifactorial process, the study of nutritional imbalances requires the use of time-consuming experimental designs and appropriate statistical and multiple regression analysis for the determination of critical parameters, whose results may be difficult to interpret when the number of variables is large. The use of machine learning (ML) supposes a cutting-edge approach to investigate multifactorial processes, with the aim of detecting non-linear relationships and critical factors affecting a determined response and their concealed interactions. Thus, in this work we applied artificial neural networks coupled to fuzzy logic, known as neurofuzzy logic, to determine the critical factors affecting the mineral nutrition of medicinal plants belonging to *Bryophyllum* subgenus cultured *in vitro*. The application of neurofuzzy logic algorithms facilitate the interpretation of the results, as the technology is able to generate useful and understandable “IF-THEN” rules, that provide information about the factor(s) involved in a certain response. In this sense, ammonium, sulfate, molybdenum, copper and sodium were the most important nutrients that explain the variation in the *in vitro* culture establishment of the medicinal plants in a species-dependent manner. Thus, our results indicate that Bryophyllum spp. display a fine-tuning regulation of mineral nutrition, that was reported for the first time under *in vitro* conditions. Overall, neurofuzzy model was able to predict and identify masked interactions among such factors, providing a source of knowledge (helpful information) from the experimental data (non-informative *per se*), in order to make the exploitation and valorization of medicinal plants with high phytochemical potential easier.

## Introduction

Recent biotechnological reports highlighted that medicinal plants constitute the source for more than the 25% of drugs officially approved by the Food and Drug Administration (FDA; Marchev et al., 2020). Furthermore, medicinal plant-derived products are effectively used in the primary healthcare systems for around the 90% of developing countries (El Sheikha, 2017). Taking this into account, the exploitation of medicinal plants has emerged as one of the major challenges in the biotechnological and pharmacological industries for this decade. Additionally, in response to current global demands, increasing efforts are being made to satisfy the future expectations of plant-derived food and drug production worldwide, thus requiring surface maximization for agricultural and nutritional purposes (Pastor et al., 2019). Consequently, novel approaches must be undertaken in the field of medicinal plant research with the aim of meeting the requirements for the large-scale exploitation of medicinal plants. In this sense, plant tissue culture (PTC) constitutes an efficient pool of methodologies becoming a sustainable platform to achieve true-to-type products with added-value properties (Eibl et al., 2018; Chandran et al., 2020) and requires much less space for achieving the same yields than conventional open field agriculture, due to their ability for scaling-up in bioreactors. This methodology confers an absolute independence of climatic threats, plant pathogens and harsh agriculture management and large storage facilities of plant materials. Nevertheless, PTC should cope with their own difficulties, as it requires the investment for specialized equipment and consumables and the recruitment of trained staff to develop the associated methodologies (Bridgen et al., 2018; Patra et al., 2020).

One of the crucial factors that impact the success of PTC establishment is the optimization of growth conditions and the mineral composition of culture medium (Isah et al., 2018). Although a high number of publications have focused on the study of *in vitro* growth conditions for many species (Batista et al., 2016; Golkar et al., 2019; Hoang et al., 2019), culture medium composition is a paramount factor usually underestimated during the design of plant *in vitro* culture protocols (Nezami-Alanagh et al., 2014; García-Pérez et al., 2020a). Under PTC conditions, the ingredients included in the culture medium constitute the only source of nutrients available for plants and subsequent nutritional imbalances may occur discretely affecting culture development (Niedz and Evens, 2007), reflecting substantial physiological symptoms (Nezami-Alanagh et al., 2019). Thus, PTC media formulations contain a wide spectrum of mineral and organic nutrients that interact in a complex, multifactorial, nonlinear and non-deterministic way, without considering the individual susceptibilities and requirements that discrete species present, leading to the existence of nutritional imbalances, causing underlying deleterious effects that may be easily detectable (Nezami-Alanagh et al., 2019; Phillips and Garda, 2019).

As a rule, culture media components contain, as average, 18 different mineral nutrients, some required at high concentrations (macronutrients) such as nitrogen, potassium, calcium, phosphorus, sulfur and magnesium, while others are required at lower concentrations (micronutrients), such as manganese, zinc, boron, molybdenum, copper and iron, among others, being all essential for certain physiological processes (Twaij et al., 2020). Together with mineral nutrients, a source of carbon, normally sucrose, as well as other organic molecules, such as vitamins and amino acids, some plant growth regulators, are supplied to media to ensure a healthy plant growth and development (Saad and Elshahed, 2012). In addition, there are additional factors that show a significant impact on mineral nutrition, such as the genotype, because even closely related species have been shown to present differential behaviors toward certain media ingredients (Gago et al., 2011; Nezami-Alanagh et al., 2014).

Due to the high heterogeneity of ingredients that make part of culture media formulations and other additional factors, such as plant genotype or growth conditions, the study of nutritional requirements applied to unknown medicinal plants leads to the design of complex multivariate experimental designs (Nezami-Alanagh et al., 2017; Teixeira da Silva et al., 2020). In the last decade (Hesami and Jones, 2020; Niazian and Niedbala, 2020), several ML algorithms have been successfully employed as alternative to traditional statistical methods and/or response surface methodology (RSM) to identify factors and interactions on complex, non-linear and non-deterministic process such as PTC (Landin et al., 2009; Gago et al., 2010a,c; Nezami-Alanagh et al., 2017). Therefore, revealing all the information encrypted over the large amount of experimental results derived from this type of multifactorial processes becomes a highly challenging task. In such cases, machine learning (ML) offers a cutting-edge computer-based methodology with the ability of handling very complex multivariate datasets, in which there are unknown patterns between inputs and outputs or large amount of uncategorized or different kind of data relating with complex processes, being able to transform data into useful information and knowledge (Gago et al., 2010c; Ertel, 2017; Bini, 2018; Freiesleben et al., 2020). On this purpose, different ML algorithms such as artificial neural networks (ANNs); deep neural networks (DNNs); convolutional neural networks (CNNs); support vector machines (SVMs) or random forest (RF) has been used in plant biotechnology (Niazian and Niedbala, 2020) and, particularly, in PTC (Gago et al., 2010a). Among all of them, ANNs have been successfully applied with the aim of establishing robust predictive models that contribute to the optimization and characterization of multifactorial processes (Landin and Rowe, 2013; Gago et al., 2014; Arteta et al., 2018; Villarrubia et al., 2018; Nezami-Alanagh et al., 2019). In addition, the combination of ANNs with fuzzy logic, the so-called neurofuzzy logic, confers several advantages in the search of critical factors that impact plant nutrition, by providing “IF-THEN” rules that make result interpretation easier, in other words, understandable for the human brain (Landin et al., 2009; Gago et al., 2010b; Gallego et al., 2011). Successful applications of neurofuzzy logic in the field of PTC for seed germination (Ayuso et al., 2017), the identification of physiological disorders associated to nutritional imbalances (Nezami-Alanagh et al., 2018, 2019), improvement of bioactive compounds accumulation (García-Pérez et al., 2020b) and revealing the role of phytohormones on plant *in vitro* organogenesis (García-Pérez et al., 2020a) have been already performed successfully.

*Bryophyllum* (genus *Kalanchoe*, Crassulaceae family) constitutes a subgenus with more than 25 plant species that have been used in the traditional medicine across both the American and African continents (Stefanowicz-Hajduk et al., 2020). Pharmacognostical and phytochemical analyses have highlighted that phenolic compounds and bufadienolides were the bioactive compounds that develop their therapeutic effects, since *Bryophyllum* spp. have been largely applied to treat infections and chronic diseases, such as diabetes, cardiovascular diseases and cancer (García-Pérez et al., 2018a). The knowledge derived from the combination of ML and PTC will be highly valuable for considering the biotechnological exploitation of *Bryophyllum* spp. in order to take advantage of their added-value properties as a potential source of bioactive compounds.

In this work, we applied the ML (ANNs algorithms), to model and provide insight about the critical factors and their interactions that drive mineral nutrition of three medicinal plants from the subgenus *Bryophyllum* cultured *in vitro*, by focusing on the content in macronutrients and micronutrients from culture media formulations, with the aim of revealing masked nutritional imbalances and interactions that may occur between nutrients that impact plant growth-related parameters.

## Materials and Methods

### Plant Material and Culture Conditions

The establishment of *in vitro* culture was conducted for three different *Bryophyllum* species, namely: *Bryophyllum daigremontianum* Raym. - Hamet et Perr. (syn. *Kalanchoe daigremontinana*, BD), *Bryophyllum* × *houghtonii* D.B. Ward (*Bryophyllum daigremontianum* × *tubiflorum*, syn. *Kalanchoe* × *houghtonii*, BH) and *Bryophyllum tubiflorum* Harv. (syn. *Kalanchoe tubiflora*, BT).

Epiphyllous plantlets from these three species were used for the disinfection and transference to *in vitro* conditions as described in previous works (García-Pérez et al., 2019). After surface disinfection, plantlets were cultured by groups of three in glass culture vessels containing 25 mL of previously autoclaved MS medium (Murashige and Skoog, 1962) supplemented with 3% sucrose and solidified with 0.8% agar at pH = 5.8. Cultures were then transferred to growth chambers and placed randomly in the shelves at 25 ± 1°C under a photoperiod of 16 h light (55 μmol m^−2^ s^−1^) and 8 h dark and subcultured every 12 weeks by using newly formed epiphyllous plantlets as the explants for next subculture.

### Experimental Design

Spontaneously rooted epiphyllous plantlets from the three *Bryophyllum* species, proceeding from 12-week-old plants grown on MS medium, were subjected to nutrition experiments. Plantlets were transferred by pairs into 10 glass culture vessels, grown and subcultured under the same conditions stated above, making a total of 20 replicates per treatment.

For nutrition experiment, nine different culture media formulations, based on MS medium were used. Due to the low mineral requirements associated to Crassulaceae plants, as it is the case of *Bryophyllum* spp. (Phillips and Garda, 2019; García-Pérez et al., 2020b), each media contained proportional reduced contents of both either MS macronutrients (M) or MS micronutrients (μ). Thus, half-concentrations (1/2MSM and 1/2MSμ), quarter-concentrations (1/4MSM and 1/4MSμ), eighth-concentrations (1/8MSM and 1/8MSμ) and macronutrient and micronutrient absence (0MSM, 0MSμ) and, as control, full MS medium was tested (Table 1). EDTA-chelated iron, vitamins and organic molecules were supplied in all media at same concentration than in the original MS formulation. All media were also supplemented with 3% sucrose and solidified with 0.8% agar at pH = 5.8.

**Table 1:** Mineral salt composition included in cultured media formulations used in this study.

	**Salt**	**MS** **(mg L^**−1**^)**	**1/2MSM** **(mg L^**−1**^)**	**1/4MSM** **(mg L^**−1**^)**	**1/8MSM** **(mg L^**−1**^)**	**0MSM** **(mg L^**−1**^)**	**1/2MSμ** **(mg L^**−1**^)**	**1/4MSμ** **(mg L^**−1**^)**	**1/8MSμ** **(mg L^**−1**^)**	**0MSμ** **(mg L^**−1**^)**
Macronutrients	KNO_3_ NH_4_NO_3_ CaCl_2_ 2H_2_O MgSO_4_ 7H_2_O KH_2_PO_4_	1,900 1650 440 370 170	950 825 220 185 85	475 412.5 110 92.5 42.5	237.5 206.3 55 46.3 21.30	0	1,900 1650 440 370 170			
Micronutrients	MnSO_4_ 4H_2_O ZnSO_4_ 7H_2_O H_3_BO_3_ KI Na_2_MoO_4_ 2H_2_O CuSO_4_ 5H_2_O CoCl_2_ 6H_2_O	22.3 8.6 6.2 0.83 0.25 0.025 0.025					11.2 4.3 3.1 0.42 0.13 0.013 0.013	5.6 2.2 1.6 0.21 0.063 0.0063 0.0063	2.8 1.1 0.78 0.10 0.031 0.0031 0.0031	0
Iron source	Na_2_EDTA FeSO_4_ 7H_2_O	37.25 27.85								

In order to observe the nutritional long-term impact on *Bryophyllum* growth parameters, four subcultures were performed. At the end of each subculture (every 12 weeks) two newly-formed epiphyllous plantlets per vessel were randomly selected, transferred to fresh medium of the same media (treatment) in which were cultured, and grown in the same conditions. In the treatments in which epiphyllous plantlet formation was not observed (such as 0MS), two rooted and newly-formed epiphyllous plantlets from the control treatment (MS) were selected and cultured in that media. In total, 20 replicates (10 vessels with two plantlets) were kept in each subculture per treatment.

Thus, the experimental design included 3 different genotypes (BD, BH and BT) × 9 different culture media formulations (MS control + 4 macronutrient-reduced formulations + 4 micronutrient-reduced formulations) × 4 subcultures, accounting for a total of 108 different treatments with 20 replicates each.

At the end of each subculture (12 weeks) six physiological parameters were determined in the new epiphyllous plantlets: shoot length (SL, expressed as cm), longest root length (RL, expressed as cm), plantlet number (PN), leaf number (LN), aerial parts fresh weight (AFW, expressed in g) and root fresh weight (RFW, expressed in g).

After experimental data collection from nutrition experiment, all data were merged into one large multifactorial database including 108 treatments following a factorial design for 18 factors (Supplementary Table 1). Salts included in culture media were split into their containing ions with the aim of avoiding ion confounding (Niedz and Evens, 2006). As a result, the eighteen factors were selected as the inputs (genotype, subculture number and 16 ions) plus the six physiological parameters as outputs (SL, RL, PN, LN, AFW, and RFW) for building the model. In all cases, results were expressed as the mean ± standard error (Supplementary Table 1).

### Statistical Analysis

Initially, data derived from the nutrition experiments (SL, RL, PN, LN, AFW, and RFW) were analyzed statistically in order to evaluate the significance of each factor and their interactions (significance level: α = 0.01) on the parameters studied. To that end, factorial ANOVA was performed to elucidate the effect of genotype, subculture and culture media and their interactions, followed by Tukey's HSD *post hoc* test (α = 0.01). Data normality and homoscedasticity was assessed by Kolmogorov-Smirnov's and Levene's tests, respectively. Count data (PN and LN) parameters should be analyzed by Poisson Regression, but as number of replicates was large (*n* = 20) and, thus, ANOVA had the same inference than Poisson Regression (α = 0.01; Mize et al., 1999). ANOVA was also applied to those parameters as in previous works (Ayuso et al., 2019). In both cases, the software used was STATISTICA v.12 (StatSoft Inc., 2014, Street Tulsa, OK, USA).

### Modeling Tools

Data modeling was performed by using the commercial FormRules® v.4.03 software (Intelligensys LTD, UK) as described elsewhere (Nezami-Alanagh et al., 2018; García-Pérez et al., 2020a). Briefly, FormRules® performed the adaptive-spline-modeling-of-data (ASMOD) algorithm to minimize the number of relevant inputs and to reduce the model complexity and facilitating accuracy with fewer parameters (Shao et al., 2006). Several statistical fitness criteria including cross validation (CV), leave one out cross validation (LOOCV), minimum description length (MDL), Bayesian information criterion (BIC) and structural risk minimization (SRM) were investigated to obtain the model that gave the best Train Set R^2^. Two of these, CV and LOOCV, split the data into subsets that are either used for training and testing (validation method), while the others (MDL, BIC and SRM) are statistical significance methods, which use all the data for training. These are designed to avoid overtraining, minimizing a criterion that contains two terms: (i) the number of parameters in the model (the variance) and (ii) the prediction errors computed on the data set (the bias). The best results were found for SRM, which ensured the highest predictability with the minimum generalization error and provided the generation of the simplest and more intelligible rules (Vapnik, 1992).

The training process was conducted as described in detail elsewhere (Shao et al., 2006) and training parameters are summarized in Table 2. The quality of submodels (predictability and accuracy), independently generated for every output, were assessed according to the ANOVA *f*-ratio, mean square error (MSE; Equation 1) and the coefficient of determination (Train Set *R*^2^; Equation 2):

(1) $$MSE=\left(\frac{\sum_{i=1}^{n}{\left(y_{i}-y_{i}^{\prime }\right)}^{2}}{n}\right)$$

(2) $$R^{2}=\left(1-\frac{\sum_{i=1}^{n}{\left(y_{i}-y_{i}^{\prime }\right)}^{2}}{\sum_{i=1}^{n}{\left(y_{i}-y_{i}^{\prime \prime }\right)}^{2}}\right)\times 100$$

Where y_i_ represents the experimental value from the data set, $y_{i}^{\prime }$ represents the predicted value by the model, and $y_{i}^{\prime \prime }$ represents the mean of the dependent variable. MSE are calculated to provide information about the random error component of the built model, thus indicating the usefulness of model fitting for prediction due to a smaller incidence of random error (Hesami and Jones, 2020). Models with high Train Set *R*^2^ (>70%) and an *f*-ratio (>4) assess model accuracy and no statistical differences among predicted and experimental values. Models with higher values than 99.9% should be rejected due to model over-fitting (Colbourn and Rowe, 2005; Landin et al., 2009).

**Table 2:** Training parameters for the construction of neurofuzzy model used by FormRules®.

**Minimization Parameters (ASMOD)**
Ridge regression factor: 1 × 10^−6^
MODEL SELECTION CRITERIA
Structural risk minimization (SRM)
C1 = 0.95, C2 = 4.8
Number of set densities: 2
Set densities: 2
Adapt nodes: TRUE
Maximum inputs per submodel: 3
Maximum nodes per input: 15

The results provided by the application of neurofuzzy logic were expressed as “IF-THEN” rules, thus making their interpretation easier, and they were given a range level (from low to high), combined with a corresponding membership degree value, that ranges between 0 and 1 (Gallego et al., 2011). Supplementary Figure 1 is attached to facilitate the understanding of the linguistic expressions of the variables obtained by the neurofuzzy logic model (Low, Medium and High).

## Results

**Figure 1:**
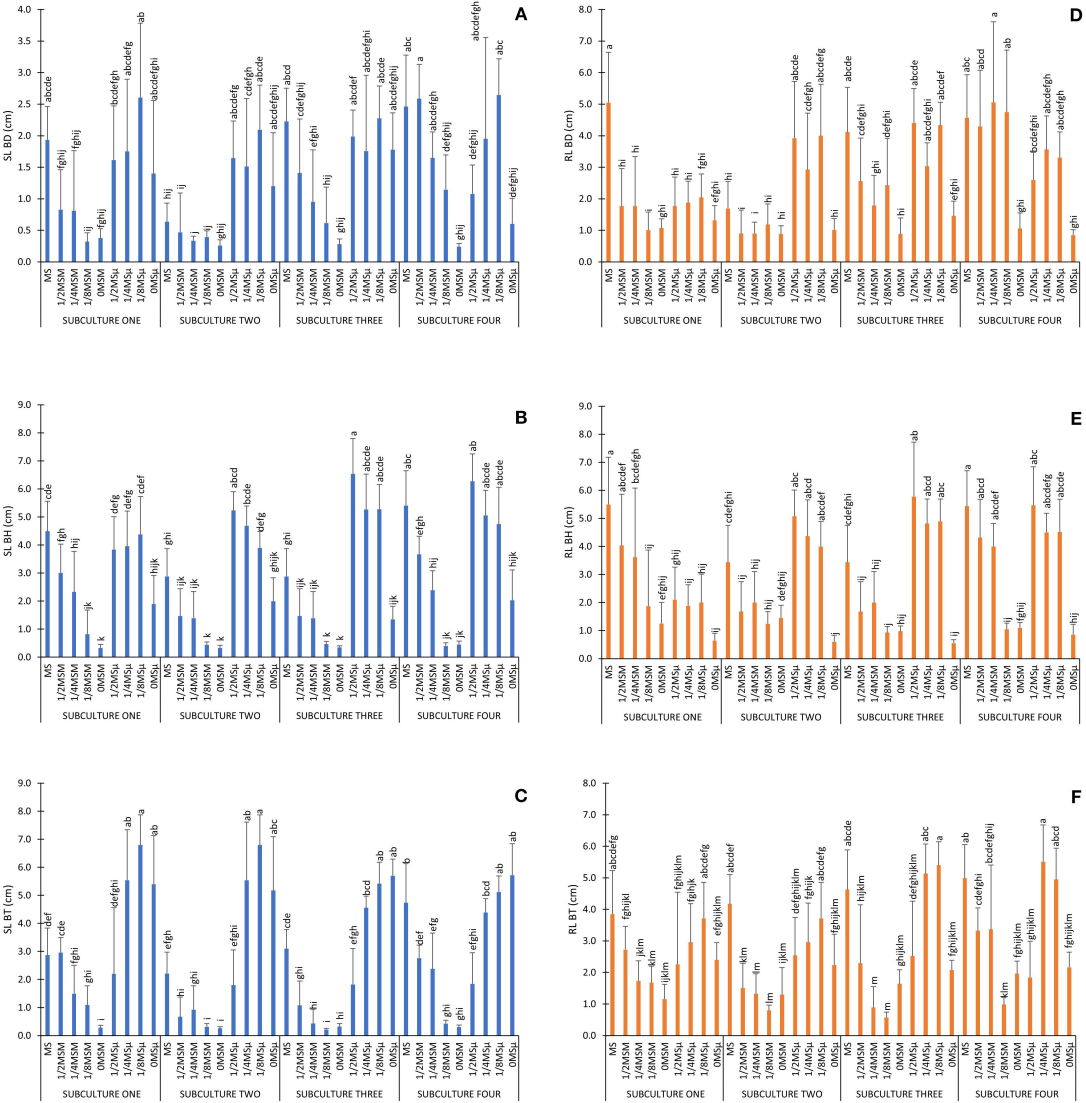
Experimental data obtained for SL and RL. Values are expressed as the mean and vertical bars indicate standard deviation. Different letters indicate significant differences (α = 0.01). **(A)** SL BD (cm); **(B)** SL BH (cm); **(C)** SL BT (cm); **(D)** RL BD (cm); **(E)** RL BH (cm); **(F)** RL BT (cm).

Traditionally plant tissue researchers used statistical method such as factorial ANOVA to analyze data and test if two or more treatment differ significantly from each other due to the effect of some independent variables (factors), but also serves to infer cause-effect relationships. Here, data analysis using factorial ANOVA reflected that all factors studied (genotype, subculture number and culture media) and all interactions among them, caused a significance effect on all parameters studied, except for the interaction genotype × subculture on RL, PN, AFW and RFW parameters (α = 0.01; Supplementary Table 2). The combined effect of the interactions between the number of subcultures and the culture medium is graphically exemplified for SL and RL in Figure 1, depending on each genotype studied: BD, BH and BT, respectively.

In general, MS full strength media promoted high SL and RL in all genotypes, although the final shoot and root length varied significantly depending on the genotype and subculture (Figure 1). Those treatments with reduced micronutrients (1/2MSμ, 1/4MSμ, and 1/8MSμ) promoted more length than those with reduced macronutrients (1/2MSM, 1/4MSM, and 1/8MSM), and the treatments with absence of minerals in the media, particularly without macronutrients (0MSM), promoted the worst results overall (Figure 1). The same effect, can be observed for the rest of parameters (see Supplementary Table 1), particularly for PN, which measures the organogenesis capacity, since the absence of mineral nutrients causes the total inhibition of the generation of new epiphyllous plantlets in the leaf margins of all species (Supplementary Table 1: treatments 9, 18, 27, 36 for BD; 45, 54, 63, 72 for BH, and 81, 90, 99, 108 for BT). All together, these ANOVA results showed that MS media composition can be modified dramatically by reducing the amount of macro and micronutrients and obtain exactly the same results. But little information was obtained about the effect of each mineral nutrient or its role on the effect observed.

Data modeling by neurofuzzy logic emerges as a solution to provide useful knowledge on *Bryophyllum* mineral nutrition after training and learning from the experimental data. The model showed high predictability with Train Set *R*^2^ values higher than 70%, *f*-ratio >4 and low values of MSE in all submodels (Table 3). In addition, model accuracy was assessed by ANOVA *f*-critical, which proved that predicted values from the model did not show statistically significant differences with respect to the experimental values for any of the outputs (α = 0.05; Table 3).

**Table 3:** Quality parameters and critical factors detected by neurofuzzy logic model.

**Output**	**Submodel**	**Significant inputs**	**MSE**	**Train Set *R*^**2**^**	***f* ratio**	**df_**1**_, df_**2**_**	***f* critical (α = 0.05)**
SL	1	${\text{NH}}_{4}^{+}$	0.92	74.97	42.79	7, 100	2.10
	**2**	**Genotype × Cu**^**2+**^					
RL	**1**	**${\text{SO}}_{4}^{2-}$ × ${\text{MoO}}_{4}^{2-}$ × Genotype**	0.74	76.97	10.91	25, 82	1.64
	2	Subculture × Na^+^					
PN	**1**	**${\text{SO}}_{4}^{2-}$ × ${\text{MoO}}_{4}^{2-}$ × Genotype**	60.60	72.85	8.76	25, 82	1.64
	2	Subculture × Na^+^					
LN	**1**	**Genotype × ${\text{NH}}_{4}^{+}$**	9.00	72.90	45.29	6, 101	2.19
AFW	**1**	**${\text{SO}}_{4}^{2-}$ × ${\text{MoO}}_{4}^{2-}$ × Genotype**	0.04	89.89	43.66	18, 89	1.72
RFW	**1**	**Genotype × Cu**^**2+**^ ** × ${\text{SO}}_{4}^{2-}$**	0.001	79.69	25.75	14, 93	1.80
	2	${\text{MoO}}_{4}^{2-}$					

*Bold inputs indicate the strongest effect associated to each output*.

For SL, the model generated two submodels being the interaction between genotype and copper the one with the highest contribution (Table 3). As previously stated, neurofuzzy model had the ability of unraveling masked interactions between different factors, once the salt media composition of each treatment was formulated as their ion composition, to avoid ion confounding. An additional submodel showed that ${\text{NH}}_{4}^{+}$ concentration also caused a significant effect but with lower contributions to SL prediction, than the interaction of genotype × Cu^2+^ (Table 3). In the case of RL, two submodels were found, being the triple interaction between the genotype, ${\text{SO}}_{4}^{2-}$ and ${\text{MoO}}_{4}^{2-}$ the one presenting the major contribution to RL. Additionally, a second submodel for RL included the interaction of number of subcultures with sodium (Table 3). Exactly the same submodels were also predicted for PN and, interestingly, this finding suggests that sulfur and molybdenum play a crucial role not only on *Bryophyllum* nutrition, but on controlling its asexual reproduction. The second submodel spotted the interaction of number of subcultures with sodium for PN, too. Concerning LN only one model was generated, being predicted by the interaction between the genotype and ${\text{NH}}_{4}^{+}$ concentration (Table 3), what indicates that this output is closely related and highly influenced by this nitrogen-containing macronutrient ion. In the case of AFW, the interaction between genotype, ${\text{SO}}_{4}^{2-}$ and ${\text{MoO}}_{4}^{2-}$ was the only factor spotted as the most significant affecting this output, in the same way than RL and PN. Finally, RFW was predicted by two submodels, showing the interaction between the genotype, Cu^2+^ and ${\text{SO}}_{4}^{2-}$ the major contribution (Table 3), being the only output that was dependent on copper besides SL. An additional submodel for RFW was predicted by molybdate concentration.

In general, the application of neurofuzzy logic identified all the significant factors on all the outputs related to *Bryophyllum in vitro* growth and their concealed interactions. Nevertheless, the information conferred by this machine learning-based tool was useful, thanks to the establishment of “IF-THEN” rules, which described how these inputs influenced their corresponding outputs. The full set of rules can be found in Supplementary Table 3, while the rules including the highest membership degrees for each output were summarized in Table 4. In order to make result interpretation easier, all factors were ranged as low, mid and high at the same time, according to their effect on every output and the experimental space tested. The graphical ranging of each ion can be visualized in Supplementary Figure 1.

**Table 4:** Summary of “IF-THEN” rules generated by ANN modeling.

**Rules**		**Genot**	**Subcult**	**${\text{NH}}_{4}^{+}$**	**${\text{SO}}_{4}^{2-}$**	**Na^**+**^**	**Cu^**2+**^**	**${\text{MoO}}_{4}^{2-}$**		**SL**	**RL**	**PN**	**LN**	**AFW**	**RFW**	**Membership**
1	I F			Low					T H E N	Low						0.98
2				High						High						0.83
**5**		**BD**					**Low**			**Low**						**1.00**
6		BD					High			Low						1.00
**7**		**BT**					**Low**			**High**						**0.77**
8		BT					High			Low						1.00
**9**	I F	**BH**			**Low**			**Low**	T H E N		**Low**					**1.00**
10		BD			Low			Low			Low					1.00
11		BT			Low			Low			Low					1.00
12		BH			Low			Mid			High					1.00
13		BD			Low			Mid			High					1.00
**14**		**BT**			**Low**			**Mid**			**High**					**1.00**
15		BH			Low			High			Low					1.00
16		BD			Low			High			Low					1.00
17		BT			Low			High			Low					1.00
18		BH			High			Low			High					1.00
19		BD			High			Low			High					1.00
20		BT			High			Low			High					1.00
21		BH			High			Mid			High					1.00
22		BD			High			Mid			High					1.00
23		BT			High			Mid			Low					1.00
24		BH			High			High			Low					1.00
25		BD			High			High			Low					1.00
26		BT			High			High			Low					1.00
27			ONE			Low					Low					1.00
28			ONE			High					High					1.00
29			TWO			Low					Low					1.00
30			TWO			High					High					1.00
31			THREE			Low					Low					1.00
32			THREE			High					High					1.00
33			FOUR			Low					Low					1.00
34			FOUR			High					High					1.00
**35**	I F	**BH**			**Low**			**Low**	T H E N			**Low**				**1.00**
36		BD			Low			Low				Low				1.00
37		BT			Low			Low				Low				1.00
38		BH			Low			Mid				Low				1.00
39		BD			Low			Mid				High				1.00
**40**		**BT**			**Low**			**Mid**				**High**				**1.00**
41		BH			Low			High				Low				1.00
42		BD			Low			High				Low				1.00
43		BT			Low			High				Low				1.00
44		BH			High			Low				High				1.00
45		BD			High			Low				High				1.00
46		BT			High			Low				High				1.00
47		BH			High			Mid				High				1.00
48		BD			High			Mid				Low				1.00
49		BT			High			Mid				Low				1.00
50		BH			High			High				Low				1.00
51		BD			High			High				Low				1.00
52		BT			High			High				Low				1.00
53			ONE			Low						Low				1.00
54			ONE			High						High				1.00
55			TWO			Low						Low				1.00
56			TWO			High						High				1.00
57			THREE			Low						Low				1.00
58			THREE			High						High				1.00
59			FOUR			Low						Low				1.00
60			FOUR			High						High				1.00
**63**	I F	**BD**		**Low**					T H E N				**Low**			**0.96**
**66**		**BT**		**High**									**High**			**0.74**
**67**	I F	**BH**			**Low**			**Low**	T H E N					**Low**		**1.00**
68		BD			Low			Low						Low		1.00
69		BT			Low			Low						Low		1.00
70		BH			Low			Mid						Low		1.00
71		BD			Low			Mid						Low		1.00
72		**BT**			**Low**			**Mid**						**High**		**1.00**
73		BH			Low			High						Low		1.00
75		BT			Low			High						Low		1.00
76		BH			High			Low						High		1.00
77		BD			High			Low						High		1.00
78		BT			High			Low						High		1.00
79		BH			High			Mid						High		1.00
80		BD			High			Mid						High		1.00
81		BT			High			Mid						Low		1.00
**85**	I F	**BH**			**Low**		**Low**		T H E N						**Low**	**1.00**
86		BH			Low		High								High	1.00
**87**		**BH**			**High**		**Low**								**High**	**1.00**
88		BH			High		High								High	1.00
89		BD			Low		Low								Low	1.00
90		BD			Low		High								High	1.00
91		BD			High		Low								High	1.00
92		BD			High		High								High	1.00
93		BT			Low		Low								Low	1.00
94		BT			Low		High								High	1.00
95		BT			High		Low								High	1.00
96		BT			High		High								High	1.00
97								Low							Low	1.00
98								Mid							Low	1.00
99								High							Low	1.00

*Inputs with the highest membership degree, showing the major contibutions for each response on every output, are indicated in bold. Only the rules with the highest membership degree were selected Genot: genotype; Subcult: number of subcultures. SL and RL were expressed in cm. AFW and RFW were expressed in g*.

As previously stated, the observed changes in the output SL was mainly caused by the interaction between genotype and Cu^2+^ and, besides that, the model generated the corresponding rules. The rules for SL indicate that high values were obtained in the case of BT when cultured under Low Cu^2+^ concentrations (<0.05 μM) with a membership degree of 0.77 (rule 7; Table 4). In fact, it was the only case that presented a High SL response related to copper. In contrast, the lowest SL value (showing a membership degree of 1.00) was obtained for BD under Low Cu^2+^ concentrations (rule 5; Table 4). Concerning the rules associated to the other submodels, High SL values were obtained under High ${\text{NH}}_{4}^{+}$ concentrations (>10.31 mM) with high membership (0.83, rule 2; Table 4). These results suggest a predominant effect of genotype and copper, over the ${\text{NH}}_{4}^{+}$, on SL.

RL, PN and AFW were predicted mainly by the interaction of three factors: genotype, ${\text{SO}}_{4}^{2-}$ and ${\text{MoO}}_{4}^{2}$. Thus, the response with the highest contribution to High RL values (membership 1.00) is the interaction between Low ${\text{SO}}_{4}^{2-}$ concentrations (<1.01 mM) with Mid ${\text{MoO}}_{4}^{2-}$ concentrations (0.25–0.75 μM) for BT (rule 14 for RL, rule 40 for PN, and rule 72 for AFW; Table 4). On the contrary, the interaction between Low ${\text{SO}}_{4}^{2-}$ concentrations (<1.01 mM) with Low ${\text{MoO}}_{4}^{2-}$ concentrations (<0.25 μM) for BH showed the highest contribution to Low RL, PN and AFW values (rules 9, 35, and 67, respectively; Table 4). In addition, RL and PN presented another submodel, based on the interaction between the number of subcultures and sodium (Table 3). In all cases, the model rules showed that High Na^+^ concentrations (0.22 mM) caused High RL and PN in all subcultures (rules 27–34 for RL, and 53–60 for PN; Table 4).

LN was exclusively predicted by the interaction between the genotype and ${\text{NH}}_{4}^{+}$ concentrations. High LN values were predicted only by High ${\text{NH}}_{4}^{+}$ concentrations for BT (>10.31 mM, membership degree 0.74, rule 66; Table 4), showing Low values for the rest of cases, specially BD at Low ${\text{NH}}_{4}^{+}$ concentrations (membership degree 0.96, rule 63; Table 4). These results suggest a predominant role of genotype, as LN was exclusively favored on BT, only if High ${\text{NH}}_{4}^{+}$ concentrations were supplied into the media.

Finally, the major submodel predicting RFW included the interaction between genotype, ${\text{SO}}_{4}^{2-}$ and Cu^2+^ concentrations. The High RFW values with the highest membership degree (1.00) were obtained for BH cultured under High ${\text{SO}}_{4}^{2-}$ concentrations (>0.88 mM) and Low Cu^2+^ concentrations (<0.05 μM, rule 87; Table 4). Meanwhile, Low values were predicted by Low concentrations of both ions (membership degree 1.00, rule 85; Table 4), independently of the genotype used (rules 85, 89 and 93; Table 4). In addition, the second submodel generated for RFW pointed at molybdate concentrations as the significant output, causing Low RFW values in all cases (rules 97–99; Table 4). These results suggest a predominant role of genotype, favored when High ${\text{SO}}_{4}^{2-}$ and/or Cu^2+^ concentrations were included into the media.

## Discussion

The combination of artificial neural networks (ANNs) with fuzzy logic, called neurofuzzy logic, constitutes ML algorithms used for predicting and identifying critical factors of multifactorial nonlinear systems (Shihabudheen and Pillai, 2017), as it is the case of plant *in vitro* nutrition (Gallego et al., 2011). Advantages of ANNs algorithms over traditional statistics have been pointed out previously (Landin et al., 2009; Gago et al., 2010a,c). In this work, the application of neurofuzzy confers a simple and efficient solution about which factors determined the effects found on each *Bryophyllum* growth parameter, by extracting the knowledge among the deep interactions learnt after data training.

Genotype was a widely distributed factor identified for the prediction of all outputs either alone or in combination with one or two additional factors. This indicates that, although the three species of the *Bryophyllum* subgenus are considered closely genetically related, each species shows different nutritional requirements, including macronutrients and micronutrients. These differences may probably be due to the transcriptional regulation of uptake systems, such as the primary response to nutrient limitation conditions, since they are highly inducible by environmental conditions (Bird, 2015). Thus, the establishment of *in vitro* culture results in an effective system to test nutritional imbalances, as it eliminates the influence of side biotic or abiotic factors that impact mineral acquisition, such as pathogen and soil-mediated interactions (Comerford, 2005; Ferrante et al., 2017). These differential patterns for *Bryophyllum* species have already been related to leaf morphology (Chernetskyy et al., 2018) and other discrepancies in physiological processes, such as the biosynthesis of phenolic compounds (Fürer et al., 2016; Bogucka-Kocka et al., 2018; García-Pérez et al., 2020a) and organogenesis (García-Pérez et al., 2020b). Furthermore, the specific growth responses predicted by the ANN model, denote that *Bryophyllum* spp. present a tight range of concentrations to achieve an efficient mineral nutrition (Shrivastav et al., 2020).

Another factor, associated to PTC technology and identified by the model as critical, was the number of subcultures. The number of subcultures was identified in combination with sodium to be significant in a secondary submodel for RL and PN. The differential number of subcultures required to achieve certain responses reveals that nutrient deficiencies may be sensed at different periods during the culture time. The delay in responses under nutritional deficiencies, may be explained as a consequence of the induced stress triggered by the increased synthesis of signaling molecules, such as nitric oxide and reactive oxygen species (ROS), mainly driven by macronutrient limitations and micronutrient limitation to a lesser degree (Hajiboland, 2012; Pérez-Pérez et al., 2012; Buet et al., 2019). Its importance relies on the decrease in the rate of epigenetic variation after successive subcultures (Smulders and de Klerk, 2011). This factor becomes crucial to assess the genetical stability of *in vitro*-cultured plants, making their valorization easier. Moreover, long-term subcultures constitute an efficient strategy to improve interesting phenomena under *in vitro* conditions, such as rooting (Mendonça et al., 2019; Wang and Yao, 2020), plant regeneration (Konar et al., 2019) and callus induction (Nakasha et al., 2016), very useful for biotechnological production of by-products from medicinal plants.

Among the nutrients, the model has identified as critical factors, the macronutrients ${\text{NH}}_{4}^{+}$ and ${\text{SO}}_{4}^{2-}$, and the micronutrients Cu^2+^, Na^+^, and ${\text{MoO}}_{4}^{2-}$. The effect of ammonia, the source of nitrogen along with nitrate in most culture media, is clear on the SL parameter. Furthermore, its effect varies depending on the genotype for LN (Table 3). Nitrogen plays a controversial role on crassulacean species, such as *Bryophyllum* spp. Differential rates of crassulacean acid metabolism (CAM) have been observed as a function of nitrogen source (Pereira et al., 2017). Thus, two groups are distinguished: nitrate-enhanced CAM species and ammonium-enhanced CAM species (Rodrigues et al., 2014). Nevertheless, there is no general rule for this classification, since different *Bryophyllum* species show particular preferences toward both nitrogen sources (Pereira and Cushman, 2019). Our results suggest that nitrogen source preferences is species-dependent. The effects caused by ${\text{NH}}_{4}^{+}$ on CAM activity are mainly negative, due to the inhibition of nocturnal transport rates of organic acids into the vacuole and the cost, in terms of energy, required for ammonium mobilization (Lüttge et al., 2000; Britto et al., 2001). However, it could be noted that such paradigm has been established for soil-grown plants and nitrogen influence may not be the same under *in vitro* conditions. In this sense, only BT presented high LN values under high ${\text{NH}}_{4}^{+}$ concentrations (rule 66), while BD and BH always showed low values, whatever the ammonium supply was within the limits of the study (rules 61–65; Supplementary Table 3). Secondarily, high SL values were obtained under high ${\text{NH}}_{4}^{+}$ concentrations (rule 2; Table 4). In addition to being an essential nutrient for plant development, ${\text{NH}}_{4}^{+}$ has recently been identified as a signal molecule that triggers both, physiological and morphological responses (Liu and von Wirén, 2017). A recent report has shown that ammonium concentration lower than 15 mM improves the biosynthesis of phenolic compounds in the aerial parts of *Bryophyllum* spp. cultured *in vitro* as a consequence of a physiological response induced by abiotic stress (García-Pérez et al., 2020a). Consequently, in order to characterize the impact of ammonium on *Bryophyllum* spp., further studies at a molecular level are required.

The model reveals a close relationship between sulfur and molybdenum, whose effects on RL, PN and AFW parameters depend on the genotype (Table 3). This close interaction between both nutrients has been already reported in other species, since molybdenum is a crucial component of molybdoenzymes, involved in sulfur metabolism, being both nutrients essential for the development of aerial tissues and roots (Mendel and Hänsch, 2002; Naqib and Jahan, 2017; Blasco et al., 2018; Bouranis et al., 2020). Furthermore, due to the identical chemical configuration of both ions, the uptake of molybdate and sulfate by roots, may take place via sulfur-specific receptors found in root tissues, thus enabling the co-absorption of molybdate with sulfate (Ali et al., 2020). This could be suggested according to the model results, since low RL, PN and AFW values were observed under low ${\text{SO}}_{4}^{2-}$ concentrations and high ${\text{MoO}}_{4}^{2-}$ concentrations, and conversely, RL, PN and AFW values were high under high ${\text{SO}}_{4}^{2-}$ concentrations and low ${\text{MoO}}_{4}^{2-}$ concentrations (Table 4). In the case of RL, sulfate plays a positive role, by promoting root biomass accumulation and nutrient uptake during root growth (Alarcón-Poblete et al., 2018), in agreement with the results describe here for RFW (Table 3). These observations indicate that sulfur is essential for *in vitro* root development on *Bryophyllum* spp., although molybdate at mid concentrations may assist to its function when sulfate is limited, as demonstrated for BT (rules 14, 40, and 72; Table 4), and reported by other authors (Alhendawi et al., 2005; Shinmachi et al., 2010). Moreover, similar results were obtained for PN.

**Figure 2:**
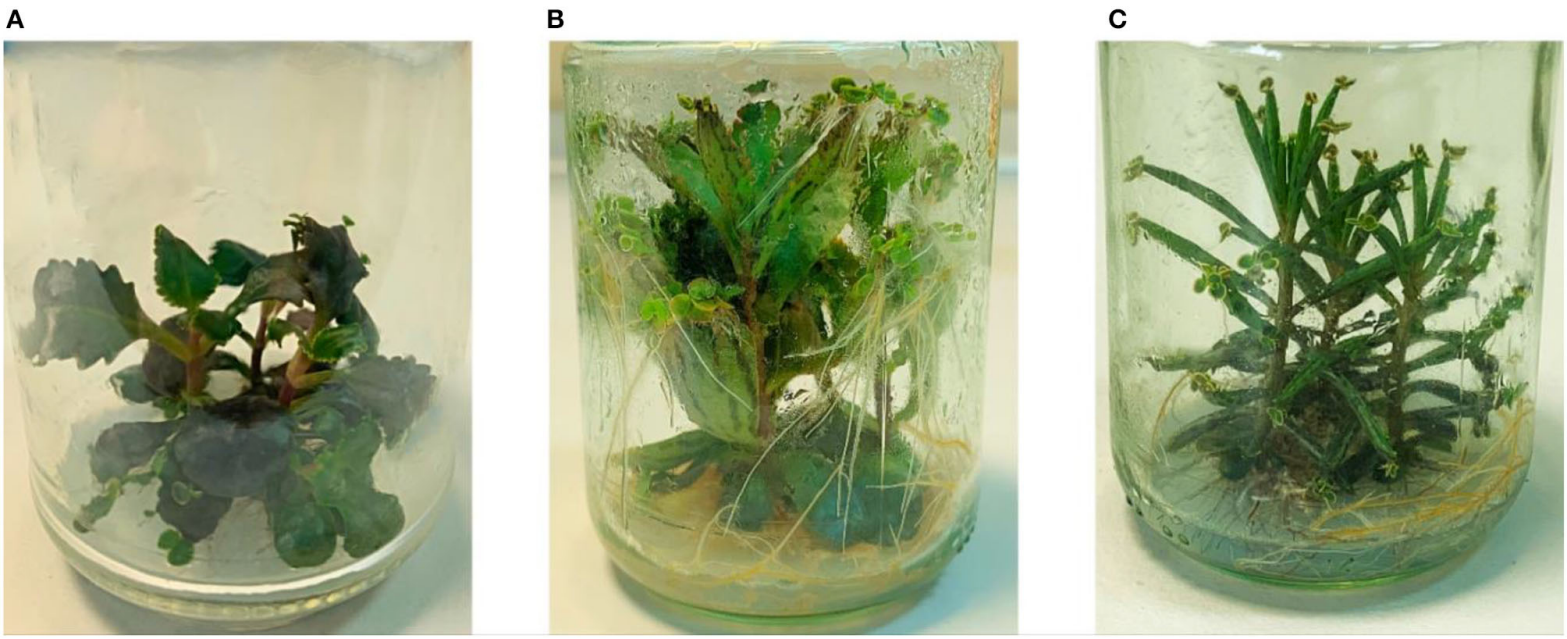
Plantlet formation in *Bryophyllum* spp. cultured *in vitro*. **(A)** Plantlets forming along the leaf margins on BD. **(B)** Plantlets forming along the leaf margins on BH. **(C)** Plantlets forming at the distal leaf end on BT.

PN was the only output associated to reproduction of *Bryophyllum* spp., since this process constitutes the mechanism developed by these species for their asexual reproduction. It combines several processes belonging to both organogenesis and embryogenesis phenomena, that have not been fully elucidated to date (Garcês et al., 2014). Such process takes place at the margin of adult leaves and is considered the major mechanism driving *Bryophyllum* clonal invasiveness, as it has been reported for BD, BH and BT (Guerra-García et al., 2015). Fully developed plantlets require the formation of their proper root systems before detaching from mother plants to form new functional clones. The development of such process may explain the close relationship between RL and PN according to their same critical factors spotted by the model (Table 3), since rooting should occur at both adult plants and newly-formed plantlets. Additionally, RL and PN presented a secondary submodel, indicating that high sodium concentrations were required for high values of both parameters from the first subculture (Table 4; >0.223 mM), which is in accordance to the sodium requirements previously reported for BT and other Crassulaceae species (George et al., 2008). In this sense, this asexual reproductive mechanism makes *Bryophyllum* spp. a suitable biological system for the establishment of *in vitro* culture, thanks to their constitutive plantlet formation. Furthermore, PN was strongly dependent on genotype, and it could be explained because of the mechanisms of plantlet formation: BH and BD develop this process along the whole leaf margins (Garcês and Sinha, 2009; Herrando-Moraira et al., 2020), whereas PN is restricted to the distal leaf end in BT (Guerra-García et al., 2018), thus potentially causing such genotype influence (Figure 2).

Copper was revealed as the most influential micronutrient on *Bryophyllum* spp. cultured *in vitro*, since it was selected as a critical factor for SL, interacting with genotype, and RFW, interacting with genotype and sulfate concentrations (Table 3). The results from the neurofuzzy logic model indicate that this nutrient affects *Bryophyllum* physiology in a species-dependent manner, as it was always found in combination with genotype. In this sense, the interaction between genotype and copper was the major factor that influenced SL. In fact, this nutrient played a differential role among the three genotypes. Only BT showed high SL values cultured under Cu^2+^ concentrations below 0.05 μM (rule 7). This indicates that BT was the genotype most affected negatively by copper, suggesting that toxicity events may occur for this species. These findings reveal that a fine-tuned control of copper homeostasis is required to prevent its toxicity due to a copper excess, since this nutrient is essential for the correct cell function, being part of highly important metalloproteins as a cofactor (Printz et al., 2016; García-Pérez et al., 2018b). Its importance relies on its contribution to basic physiological processes in plants, such as early plant growth, photosynthetic efficiency, mitochondrial respiration and the impairment of oxidative stress (Schulten and Kramer, 2017; Blasco et al., 2018). This wide influence on plant physiology could aid explaining why this nutrient was crucial for SL, associated to aerial part development, SL, and linked to root formation and growth, RFW. Such hypothesis is reinforced by the sophisticated mechanism of copper distribution within plant tissues that contains preventive molecular mechanisms enabling its accumulation by preventing eventual toxic effects at root level (Castro et al., 2018; Migocka and Malas, 2018). In the case of RFW, the coordinate action of sulfate with copper, as described by the ML model (Table 3), may indicate that sulfur contributes to such copper-induced toxic prevention, as it was earlier stated to other metals. In addition, RFW presented a second submodel that included molybdate as a critical factor, that was shown as a negative factor on this parameter, according to the model rules (rules 97–99). This observation can be justified by the effects reported for molybdenum excess, including a severe impairment of photosynthetic efficiency and the inhibition of rooting in other species (Arif et al., 2016). Thus, our results suggest that a minimum copper supplementation (<0.05 μM) may efficiently contribute to *in vitro-*cultured *Bryophyllum* plant growth.

In conclusion, our results show that the lack of specific culture media forces the use of universal formulations, as MS medium. Although such formulations contain complete combinations of mineral essential nutrients, may suppose supra-optimal concentrations for the cultivation of many species (Phillips and Garda, 2019), particularly for little-studied medicinal plants with high phytochemical potential. The nutritional imbalances spotted by ML offered a source of knowledge for the prediction of critical factors affecting *Bryophyllum* spp. plant *in vitro* culture. Through the ion split approach, neurofuzzy logic model was able to shed light on the masked interactions that take place during the *in vitro* culture of three different *Bryophyllum* species, by additionally highlighting the importance of related factors, such as the genotype and the number of subcultures. The essentiality of achieving an enhanced nutritional profiling for the correct development of medicinal plants, as it is the case of *Bryophyllum* spp., is a paramount feature that should be successfully accomplished in order to get a sustainable exploitation of such species with the aim of reaching large-scale applications in several fields, such as food, cosmetics and nutraceutical industries.

## Data Availability Statement

The original contributions presented in the study are included in the article/Supplementary Materials, further inquiries can be directed to the corresponding author/s.

## Author Contributions

PG-P and EL-M performed the experiments. PG contributed with reagents and materials. PG-P, ML, and PG contributed with modeling and analysis tools. PG-P and PG conceived the experimental design and wrote the manuscript. All authors contributed in the revision and acceptance version of the manuscript.

## Conflict of Interest

The authors declare that the research was conducted in the absence of any commercial or financial relationships that could be construed as a potential conflict of interest.
