# Supplementary material for: Machine Learning Unmasked Nutritional Imbalances on the Medicinal Plant Bryophyllum sp. Cultured in vitro
Source: Front Plant Sci. 2020 Dec 1;11:576177. doi: 10.3389/fpls.2020.576177 (PMC7729169; doi:10.3389/fpls.2020.576177)
Supplement: Supplementary file 1 [file Table_1.DOCX]

Supplementary Material

Table S1. Dataset used for modelling including the 108 treatments with their corresponding experimental results for nutrition experiments. Results are expressed as mean ± standard error (n=20). “Treat.” stands for treatment; “Genot.” stands for genotype; “Subc.” stands for subculture. SL and RL were expressed in cm, AFW and RFW were expressed in g.

| **Treat.** | **Genot.** | **Subc.** | **NH_4_^+^** | **NO_3_^-^** | **K^+^** | **Ca^2+^** | **Mg^2+^** | **HPO_4_^2-^** | **SO_4_^-2^** | **Cl^-^** | **BO_3_^3-^** | **Mn^2+^** | **Zn^2+^** | **Cu^2+^** | **MoO_4_^2-^** | **Na^+^** | **Co^2+^** | **I^-^** | **SL** | **RL** | **PN** | **LN** | **AFW** | **RFW** |
| --- | --- | --- | --- | --- | --- | --- | --- | --- | --- | --- | --- | --- | --- | --- | --- | --- | --- | --- | --- | --- | --- | --- | --- | --- |
| 1 | BD | ONE | 20.61 | 39.41 | 20.05 | 2.99 | 1.50 | 1.25 | 1.76 | 5.99 | 0.100 | 0.132 | 0.030 | 1E-04 | 0.001 | 0.2239 | 1E-04 | 0.005 | 1.9±0.1 | 5.1±0.4 | 16.4±0.8 | 5.2±0.2 | 0.63±0.04 | 0.09±0.01 |
| 2 | BD | ONE | 10.31 | 19.70 | 10.03 | 1.50 | 0.75 | 0.62 | 1.01 | 2.99 | 0.100 | 0.132 | 0.030 | 1E-04 | 0.001 | 0.2239 | 1E-04 | 0.005 | 0.8±0.1 | 1.8±0.3 | 10.2±2.3 | 3.9±0.3 | 0.25±0.07 | 0.04±0.01 |
| 3 | BD | ONE | 5.15 | 9.85 | 5.02 | 0.75 | 0.38 | 0.31 | 0.64 | 1.50 | 0.100 | 0.132 | 0.030 | 1E-04 | 0.001 | 0.2239 | 1E-04 | 0.005 | 0.8±0.2 | 1.8±0.4 | 9.6±1.9 | 3.7±0.5 | 0.20±0.08 | 0.03±0.01 |
| 4 | BD | ONE | 2.58 | 4.93 | 2.51 | 0.37 | 0.19 | 0.16 | 0.45 | 0.75 | 0.100 | 0.132 | 0.030 | 1E-04 | 0.001 | 0.2239 | 1E-04 | 0.005 | 0.3±0.03 | 1.0±0.1 | 0.8±0.3 | 3.5±0.2 | 0.04±0.03 | 0.01±0.001 |
| 5 | BD | ONE | 0.0 | 0.0 | 0.0 | 0.0 | 0.0 | 0.0 | 0.26 | 0.0 | 0.100 | 0.132 | 0.030 | 1E-04 | 0.001 | 0.2239 | 1E-04 | 0.005 | 0.3±0.03 | 0.9±0.1 | 0.2±0.1 | 2.4±0.2 | 0.01±0.001 | 0.002±0.001 |
| 6 | BD | ONE | 20.61 | 39.41 | 20.04 | 2.99 | 1.50 | 1.25 | 1.68 | 5.99 | 0.050 | 0.066 | 0.015 | 5.0E-05 | 5.0E-04 | 0.2229 | 5.3E-05 | 0.0025 | 1.6±0.2 | 1.8±0.2 | 1.6±0.4 | 5.5±0.4 | 0.43±0.08 | 0.05±0.01 |
| 7 | BD | ONE | 20.61 | 39.41 | 20.04 | 2.99 | 1.50 | 1.25 | 1.64 | 5.99 | 0.025 | 0.033 | 0.007 | 2.5E-05 | 2.5E-04 | 0.2224 | 2.6E-05 | 0.0012 | 1.8±0.3 | 1.9±0.2 | 3.1±0.5 | 6.3±0.4 | 0.38±0.08 | 0.05±0.01 |
| 8 | BD | ONE | 20.61 | 39.41 | 20.04 | 2.99 | 1.50 | 1.25 | 1.62 | 5.99 | 0.013 | 0.016 | 0.004 | 1.3E-05 | 1.3E-04 | 0.2221 | 1.3E-05 | 6.0E-04 | 2.6±0.3 | 2.0±0.2 | 2.8±0.6 | 6.2±0.6 | 0.53±0.09 | 0.04±0.01 |
| 9 | BD | ONE | 20.61 | 39.41 | 20.04 | 2.99 | 1.50 | 1.25 | 1.60 | 5.99 | 0.0 | 0.0 | 0.0 | 0.0 | 0.0 | 0.2219 | 0.0 | 0.0 | 1.4±0.2 | 1.3±0.1 | 0.0±0.0 | 4.8±0.6 | 0.37±0.08 | 0.007±0.001 |
| 10 | BD | TWO | 20.61 | 39.41 | 20.05 | 2.99 | 1.50 | 1.25 | 1.76 | 5.99 | 0.100 | 0.132 | 0.030 | 1E-04 | 0.001 | 0.2239 | 1E-04 | 0.005 | 0.6±0.1 | 1.7±0.2 | 5.8±0.7 | 3.9±0.3 | 0.10±0.02 | 0.02±0.002 |
| 11 | BD | TWO | 10.31 | 19.70 | 10.03 | 1.50 | 0.75 | 0.62 | 1.01 | 2.99 | 0.100 | 0.132 | 0.030 | 1E-04 | 0.001 | 0.2239 | 1E-04 | 0.005 | 0.5±0.1 | 0.9±0.2 | 1.0±0.6 | 2.4±0.3 | 0.07±0.004 | 0.01±0.004 |
| 12 | BD | TWO | 5.15 | 9.85 | 5.02 | 0.75 | 0.38 | 0.31 | 0.64 | 1.50 | 0.100 | 0.132 | 0.030 | 1E-04 | 0.001 | 0.2239 | 1E-04 | 0.005 | 0.3±0.02 | 0.9±0.1 | 0.6±0.2 | 2.0±0.0 | 0.02±0.003 | 0.004±0.001 |
| 13 | BD | TWO | 2.58 | 4.93 | 2.51 | 0.37 | 0.19 | 0.16 | 0.45 | 0.75 | 0.100 | 0.132 | 0.030 | 1E-04 | 0.001 | 0.2239 | 1E-04 | 0.005 | 0.4±0.03 | 1.2±0.2 | 1.4±0.6 | 2.0±0.0 | 0.02±0.003 | 0.01±0.001 |
| 14 | BD | TWO | 0.0 | 0.0 | 0.0 | 0.0 | 0.0 | 0.0 | 0.26 | 0.0 | 0.100 | 0.132 | 0.030 | 1E-04 | 0.001 | 0.2239 | 1E-04 | 0.005 | 0.3±0.02 | 1.2±0.1 | 0.2±0.1 | 2.4±0.2 | 0.02±0.004 | 0.004±0.001 |
| 15 | BD | TWO | 20.61 | 39.41 | 20.04 | 2.99 | 1.50 | 1.25 | 1.68 | 5.99 | 0.050 | 0.066 | 0.015 | 5.0E-05 | 5.0E-04 | 0.2229 | 5.3E-05 | 0.0025 | 1.6±0.1 | 3.9±0.4 | 11.2±0.9 | 6.5±0.3 | 0.44±0.04 | 0.05±0.004 |
| 16 | BD | TWO | 20.61 | 39.41 | 20.04 | 2.99 | 1.50 | 1.25 | 1.64 | 5.99 | 0.025 | 0.033 | 0.007 | 2.5E-05 | 2.5E-04 | 0.2224 | 2.6E-05 | 0.0012 | 1.5±0.3 | 2.9±0.4 | 10.7±2.2 | 5.8±0.6 | 0.52±0.15 | 0.05±0.01 |
| 17 | BD | TWO | 20.61 | 39.41 | 20.04 | 2.99 | 1.50 | 1.25 | 1.62 | 5.99 | 0.013 | 0.016 | 0.004 | 1.3E-05 | 1.3E-04 | 0.2221 | 1.3E-05 | 6.0E-04 | 2.1±0.2 | 4.0±0.5 | 12.0±2.3 | 8.3±0.3 | 0.78±0.10 | 0.08±0.01 |
| 18 | BD | TWO | 20.61 | 39.41 | 20.04 | 2.99 | 1.50 | 1.25 | 1.60 | 5.99 | 0.0 | 0.0 | 0.0 | 0.0 | 0.0 | 0.2219 | 0.0 | 0.0 | 1.2±0.2 | 1.0±0.1 | 0.0±0.0 | 5.2±0.5 | 0.21±0.04 | 0.004±0.001 |
| 19 | BD | THREE | 20.61 | 39.41 | 20.05 | 2.99 | 1.50 | 1.25 | 1.76 | 5.99 | 0.100 | 0.132 | 0.030 | 1E-04 | 0.001 | 0.2239 | 1E-04 | 0.005 | 2.2±0.1 | 4.1±0.4 | 18.4±2.1 | 6.7±0.3 | 0.68±0.09 | 0.08±0.01 |
| 20 | BD | THREE | 10.31 | 19.70 | 10.03 | 1.50 | 0.75 | 0.62 | 1.01 | 2.99 | 0.100 | 0.132 | 0.030 | 1E-04 | 0.001 | 0.2239 | 1E-04 | 0.005 | 1.4±0.2 | 2.6±0.4 | 16.1±3.1 | 5.5±0.4 | 0.31±0.07 | 0.05±0.01 |
| 21 | BD | THREE | 5.15 | 9.85 | 5.02 | 0.75 | 0.38 | 0.31 | 0.64 | 1.50 | 0.100 | 0.132 | 0.030 | 1E-04 | 0.001 | 0.2239 | 1E-04 | 0.005 | 1.0±0.2 | 1.8±0.3 | 8.5±2.6 | 3.4±0.5 | 0.12±0.04 | 0.04±0.01 |
| 22 | BD | THREE | 2.58 | 4.93 | 2.51 | 0.37 | 0.19 | 0.16 | 0.45 | 0.75 | 0.100 | 0.132 | 0.030 | 1E-04 | 0.001 | 0.2239 | 1E-04 | 0.005 | 0.6±0.1 | 2.4±0.4 | 5.5±1.6 | 2.6±0.2 | 0.09±0.03 | 0.02±0.01 |
| 23 | BD | THREE | 0.0 | 0.0 | 0.0 | 0.0 | 0.0 | 0.0 | 0.26 | 0.0 | 0.100 | 0.132 | 0.030 | 1E-04 | 0.001 | 0.2239 | 1E-04 | 0.005 | 0.3±0.02 | 0.9±0.1 | 0.0±0.0 | 2.5±0.2 | 0.01±0.001 | 0.002±0.001 |
| 24 | BD | THREE | 20.61 | 39.41 | 20.04 | 2.99 | 1.50 | 1.25 | 1.68 | 5.99 | 0.050 | 0.066 | 0.015 | 5.0E-05 | 5.0E-04 | 0.2229 | 5.3E-05 | 0.0025 | 2.0±0.1 | 4.4±0.4 | 9.9±1.0 | 6.7±0.3 | 0.52±0.05 | 0.05±0.01 |
| 25 | BD | THREE | 20.61 | 39.41 | 20.04 | 2.99 | 1.50 | 1.25 | 1.64 | 5.99 | 0.025 | 0.033 | 0.007 | 2.5E-05 | 2.5E-04 | 0.2224 | 2.6E-05 | 0.0012 | 1.8±0.2 | 3.0±0.1 | 5.9±0.4 | 6.3±0.1 | 0.42±0.04 | 0.04±0.001 |
| 26 | BD | THREE | 20.61 | 39.41 | 20.04 | 2.99 | 1.50 | 1.25 | 1.62 | 5.99 | 0.013 | 0.016 | 0.004 | 1.3E-05 | 1.3E-04 | 0.2221 | 1.3E-05 | 6.0E-04 | 2.3±0.2 | 4.3±0.2 | 13.7±1.8 | 7.6±0.3 | 0.70±0.02 | 0.06±0.01 |
| 27 | BD | THREE | 20.61 | 39.41 | 20.04 | 2.99 | 1.50 | 1.25 | 1.60 | 5.99 | 0.0 | 0.0 | 0.0 | 0.0 | 0.0 | 0.2219 | 0.0 | 0.0 | 1.8±0.1 | 1.5±0.1 | 0.0±0.0 | 6.4±0.3 | 0.40±0.05 | 0.01±0.001 |
| 28 | BD | FOUR | 20.61 | 39.41 | 20.05 | 2.99 | 1.50 | 1.25 | 1.76 | 5.99 | 0.100 | 0.132 | 0.030 | 1E-04 | 0.001 | 0.2239 | 1E-04 | 0.005 | 2.5±0.2 | 4.6±0.3 | 10.6±2.0 | 7.3±0.3 | 0.71±0.09 | 0.05±0.01 |
| 29 | BD | FOUR | 10.31 | 19.70 | 10.03 | 1.50 | 0.75 | 0.62 | 1.01 | 2.99 | 0.100 | 0.132 | 0.030 | 1E-04 | 0.001 | 0.2239 | 1E-04 | 0.005 | 2.6±0.1 | 4.3±0.4 | 25.3±2.2 | 8.3±0.3 | 0.84±0.08 | 0.06±0.01 |
| 30 | BD | FOUR | 5.15 | 9.85 | 5.02 | 0.75 | 0.38 | 0.31 | 0.64 | 1.50 | 0.100 | 0.132 | 0.030 | 1E-04 | 0.001 | 0.2239 | 1E-04 | 0.005 | 1.7±0.1 | 5.1±0.6 | 21.7±1.9 | 6.1±0.3 | 0.48±0.04 | 0.06±0.01 |
| 31 | BD | FOUR | 2.58 | 4.93 | 2.51 | 0.37 | 0.19 | 0.16 | 0.45 | 0.75 | 0.100 | 0.132 | 0.030 | 1E-04 | 0.001 | 0.2239 | 1E-04 | 0.005 | 1.1±0.1 | 4.7±0.5 | 11.4±1.4 | 4.2±0.3 | 0.25±0.03 | 0.03±0.003 |
| 32 | BD | FOUR | 0.0 | 0.0 | 0.0 | 0.0 | 0.0 | 0.0 | 0.26 | 0.0 | 0.100 | 0.132 | 0.030 | 1E-04 | 0.001 | 0.2239 | 1E-04 | 0.005 | 0.3±0.02 | 0.9±0.1 | 0.0±0.0 | 2.0±0.0 | 0.01±0.001 | 0.002±0.001 |
| 33 | BD | FOUR | 20.61 | 39.41 | 20.04 | 2.99 | 1.50 | 1.25 | 1.68 | 5.99 | 0.050 | 0.066 | 0.015 | 5.0E-05 | 5.0E-04 | 0.2229 | 5.3E-05 | 0.0025 | 1.1±0.2 | 2.6±0.3 | 7.9±0.8 | 5.3±0.3 | 0.27±0.05 | 0.03±0.005 |
| 34 | BD | FOUR | 20.61 | 39.41 | 20.04 | 2.99 | 1.50 | 1.25 | 1.64 | 5.99 | 0.025 | 0.033 | 0.007 | 2.5E-05 | 2.5E-04 | 0.2224 | 2.6E-05 | 0.0012 | 2.0±0.3 | 3.6±0.4 | 9.1±0.8 | 7.0±0.5 | 0.54±0.16 | 0.04±0.01 |
| 35 | BD | FOUR | 20.61 | 39.41 | 20.04 | 2.99 | 1.50 | 1.25 | 1.62 | 5.99 | 0.013 | 0.016 | 0.004 | 1.3E-05 | 1.3E-04 | 0.2221 | 1.3E-05 | 6.0E-04 | 2.6±0.2 | 3.3±0.3 | 12.3±1.4 | 6.7±0.3 | 0.73±0.04 | 0.08±0.01 |
| 36 | BD | FOUR | 20.61 | 39.41 | 20.04 | 2.99 | 1.50 | 1.25 | 1.60 | 5.99 | 0.0 | 0.0 | 0.0 | 0.0 | 0.0 | 0.2219 | 0.0 | 0.0 | 0.6±0.1 | 0.9±0.04 | 0.0±0.0 | 2.5±0.3 | 0.05±0.01 | 0.003±0.001 |
| 37 | BH | ONE | 20.61 | 39.41 | 20.05 | 2.99 | 1.50 | 1.25 | 1.76 | 5.99 | 0.100 | 0.132 | 0.030 | 1E-04 | 0.001 | 0.2239 | 1E-04 | 0.005 | 4.5±0.2 | 5.5±0.4 | 32.1±2.4 | 11.2±0.3 | 2.28±0.11 | 0.32±0.01 |
| 38 | BH | ONE | 10.31 | 19.70 | 10.03 | 1.50 | 0.75 | 0.62 | 1.01 | 2.99 | 0.100 | 0.132 | 0.030 | 1E-04 | 0.001 | 0.2239 | 1E-04 | 0.005 | 3.0±0.2 | 4.0±0.4 | 21.6±4.2 | 9.4±0.4 | 1.01±0.11 | 0.21±0.02 |
| 39 | BH | ONE | 5.15 | 9.85 | 5.02 | 0.75 | 0.38 | 0.31 | 0.64 | 1.50 | 0.100 | 0.132 | 0.030 | 1E-04 | 0.001 | 0.2239 | 1E-04 | 0.005 | 2.3±0.3 | 3.6±0.5 | 18.9±3.7 | 8.1±0.6 | 0.60±0.10 | 0.11±0.02 |
| 40 | BH | ONE | 2.58 | 4.93 | 2.51 | 0.37 | 0.19 | 0.16 | 0.45 | 0.75 | 0.100 | 0.132 | 0.030 | 1E-04 | 0.001 | 0.2239 | 1E-04 | 0.005 | 0.8±0.2 | 1.9±0.4 | 4.3±2.0 | 4.5±0.5 | 0.15±0.05 | 0.03±0.01 |
| 41 | BH | ONE | 0.0 | 0.0 | 0.0 | 0.0 | 0.0 | 0.0 | 0.26 | 0.0 | 0.100 | 0.132 | 0.030 | 1E-04 | 0.001 | 0.2239 | 1E-04 | 0.005 | 0.3±0.03 | 1.3±0.2 | 0.0±0.0 | 2.0±0.0 | 0.02±0.003 | 0.002±0.001 |
| 42 | BH | ONE | 20.61 | 39.41 | 20.04 | 2.99 | 1.50 | 1.25 | 1.68 | 5.99 | 0.050 | 0.066 | 0.015 | 5.0E-05 | 5.0E-04 | 0.2229 | 5.3E-05 | 0.0025 | 3.8±0.3 | 2.1±0.3 | 3.5±1.1 | 9.4±0.5 | 1.43±0.16 | 0.19±0.01 |
| 43 | BH | ONE | 20.61 | 39.41 | 20.04 | 2.99 | 1.50 | 1.25 | 1.64 | 5.99 | 0.025 | 0.033 | 0.007 | 2.5E-05 | 2.5E-04 | 0.2224 | 2.6E-05 | 0.0012 | 4.0±0.3 | 1.9±0.2 | 6.5±2.3 | 10.1±0.5 | 1.56±0.16 | 0.14±0.01 |
| 44 | BH | ONE | 20.61 | 39.41 | 20.04 | 2.99 | 1.50 | 1.25 | 1.62 | 5.99 | 0.013 | 0.016 | 0.004 | 1.3E-05 | 1.3E-04 | 0.2221 | 1.3E-05 | 6.0E-04 | 4.4±0.3 | 2.0±0.2 | 4.0±2.1 | 10.2±0.6 | 1.65±0.19 | 0.08±0.01 |
| 45 | BH | ONE | 20.61 | 39.41 | 20.04 | 2.99 | 1.50 | 1.25 | 1.60 | 5.99 | 0.0 | 0.0 | 0.0 | 0.0 | 0.0 | 0.2219 | 0.0 | 0.0 | 1.9±0.5 | 0.6±0.1 | 0.0±0.0 | 8.0±0.6 | 0.53±0.11 | 0.005±0.001 |
| 46 | BH | TWO | 20.61 | 39.41 | 20.05 | 2.99 | 1.50 | 1.25 | 1.76 | 5.99 | 0.100 | 0.132 | 0.030 | 1E-04 | 0.001 | 0.2239 | 1E-04 | 0.005 | 3.2±0.3 | 4.4±0.3 | 15.1±2.8 | 10.0±0.5 | 1.30±0.15 | 0.19±0.02 |
| 47 | BH | TWO | 10.31 | 19.70 | 10.03 | 1.50 | 0.75 | 0.62 | 1.01 | 2.99 | 0.100 | 0.132 | 0.030 | 1E-04 | 0.001 | 0.2239 | 1E-04 | 0.005 | 2.1±0.4 | 2.9±0.6 | 10.7±3.2 | 8.2±0.9 | 0.68±0.18 | 0.13±0.02 |
| 48 | BH | TWO | 5.15 | 9.85 | 5.02 | 0.75 | 0.38 | 0.31 | 0.64 | 1.50 | 0.100 | 0.132 | 0.030 | 1E-04 | 0.001 | 0.2239 | 1E-04 | 0.005 | 1.4±0.3 | 2.2±0.4 | 9.9±3.5 | 5.5±0.9 | 0.29±0.08 | 0.06±0.02 |
| 49 | BH | TWO | 2.58 | 4.93 | 2.51 | 0.37 | 0.19 | 0.16 | 0.45 | 0.75 | 0.100 | 0.132 | 0.030 | 1E-04 | 0.001 | 0.2239 | 1E-04 | 0.005 | 0.5±0.02 | 1.4±0.1 | 0.0±0.0 | 2.0±0.0 | 0.03±0.001 | 0.003±0.001 |
| 50 | BH | TWO | 0.0 | 0.0 | 0.0 | 0.0 | 0.0 | 0.0 | 0.26 | 0.0 | 0.100 | 0.132 | 0.030 | 1E-04 | 0.001 | 0.2239 | 1E-04 | 0.005 | 0.3±0.02 | 1.5±0.1 | 0.0±0.0 | 2.0±0.0 | 0.02±0.001 | 0.002±0.001 |
| 51 | BH | TWO | 20.61 | 39.41 | 20.04 | 2.99 | 1.50 | 1.25 | 1.68 | 5.99 | 0.050 | 0.066 | 0.015 | 5.0E-05 | 5.0E-04 | 0.2229 | 5.3E-05 | 0.0025 | 5.2±0.2 | 5.1±0.2 | 30.1±2.0 | 10.9±0.3 | 2.04±0.11 | 0.019±0.01 |
| 52 | BH | TWO | 20.61 | 39.41 | 20.04 | 2.99 | 1.50 | 1.25 | 1.64 | 5.99 | 0.025 | 0.033 | 0.007 | 2.5E-05 | 2.5E-04 | 0.2224 | 2.6E-05 | 0.0012 | 4.7±0.2 | 4.4±0.3 | 27.6±2.2 | 11.1±0.2 | 1.81±0.09 | 0.15±0.01 |
| 53 | BH | TWO | 20.61 | 39.41 | 20.04 | 2.99 | 1.50 | 1.25 | 1.62 | 5.99 | 0.013 | 0.016 | 0.004 | 1.3E-05 | 1.3E-04 | 0.2221 | 1.3E-05 | 6.0E-04 | 3.9±0.2 | 4.0±0.2 | 26.5±1.6 | 10.1±0.4 | 1.43±0.08 | 0.13±0.01 |
| 54 | BH | TWO | 20.61 | 39.41 | 20.04 | 2.99 | 1.50 | 1.25 | 1.60 | 5.99 | 0.0 | 0.0 | 0.0 | 0.0 | 0.0 | 0.2219 | 0.0 | 0.0 | 2.0±0.4 | 0.6±0.1 | 0.0±0.0 | 7.2±0.5 | 0.54±0.09 | 0.01±0.002 |
| 55 | BH | THREE | 20.61 | 39.41 | 20.05 | 2.99 | 1.50 | 1.25 | 1.76 | 5.99 | 0.100 | 0.132 | 0.030 | 1E-04 | 0.001 | 0.2239 | 1E-04 | 0.005 | 2.9±0.2 | 3.4±0.3 | 8.3±2.0 | 6.8±0.4 | 1.03±0.13 | 0.19±0.01 |
| 56 | BH | THREE | 10.31 | 19.70 | 10.03 | 1.50 | 0.75 | 0.62 | 1.01 | 2.99 | 0.100 | 0.132 | 0.030 | 1E-04 | 0.001 | 0.2239 | 1E-04 | 0.005 | 1.5±0.2 | 1.7±0.3 | 4.9±2.3 | 5.3±0.6 | 0.34±0.08 | 0.07±0.02 |
| 57 | BH | THREE | 5.15 | 9.85 | 5.02 | 0.75 | 0.38 | 0.31 | 0.64 | 1.50 | 0.100 | 0.132 | 0.030 | 1E-04 | 0.001 | 0.2239 | 1E-04 | 0.005 | 1.4±0.2 | 2.0±0.3 | 7.4±2.0 | 5.0±0.6 | 0.24±0.05 | 0.04±0.01 |
| 58 | BH | THREE | 2.58 | 4.93 | 2.51 | 0.37 | 0.19 | 0.16 | 0.45 | 0.75 | 0.100 | 0.132 | 0.030 | 1E-04 | 0.001 | 0.2239 | 1E-04 | 0.005 | 0.5±0.01 | 1.0±0.05 | 0.0±0.0 | 2.0±0.0 | 0.03±0.001 | 0.002±0.001 |
| 59 | BH | THREE | 0.0 | 0.0 | 0.0 | 0.0 | 0.0 | 0.0 | 0.26 | 0.0 | 0.100 | 0.132 | 0.030 | 1E-04 | 0.001 | 0.2239 | 1E-04 | 0.005 | 0.4±0.01 | 1.0±0.05 | 0.0±0.0 | 2.0±0.0 | 0.02±0.001 | 0.002±0.001 |
| 60 | BH | THREE | 20.61 | 39.41 | 20.04 | 2.99 | 1.50 | 1.25 | 1.68 | 5.99 | 0.050 | 0.066 | 0.015 | 5.0E-05 | 5.0E-04 | 0.2229 | 5.3E-05 | 0.0025 | 6.5±0.4 | 5.8±0.6 | 44.9±2.0 | 12.0±0.3 | 2.00±0.17 | 0.18±0.02 |
| 61 | BH | THREE | 20.61 | 39.41 | 20.04 | 2.99 | 1.50 | 1.25 | 1.64 | 5.99 | 0.025 | 0.033 | 0.007 | 2.5E-05 | 2.5E-04 | 0.2224 | 2.6E-05 | 0.0012 | 5.5±0.4 | 4.8±0.3 | 35.7±2.7 | 11.4±0.6 | 1.90±0.21 | 0.30±0.02 |
| 62 | BH | THREE | 20.61 | 39.41 | 20.04 | 2.99 | 1.50 | 1.25 | 1.62 | 5.99 | 0.013 | 0.016 | 0.004 | 1.3E-05 | 1.3E-04 | 0.2221 | 1.3E-05 | 6.0E-04 | 5.3±0.3 | 4.9±0.3 | 36.9±2.4 | 12.2±0.2 | 1.87±0.10 | 0.22±0.02 |
| 63 | BH | THREE | 20.61 | 39.41 | 20.04 | 2.99 | 1.50 | 1.25 | 1.60 | 5.99 | 0.0 | 0.0 | 0.0 | 0.0 | 0.0 | 0.2219 | 0.0 | 0.0 | 1.4±0.2 | 0.6±0.1 | 0.0±0.0 | 5.5±0.5 | 0.30±0.05 | 0.004±0.002 |
| 64 | BH | FOUR | 20.61 | 39.41 | 20.05 | 2.99 | 1.50 | 1.25 | 1.76 | 5.99 | 0.100 | 0.132 | 0.030 | 1E-04 | 0.001 | 0.2239 | 1E-04 | 0.005 | 5.4±0.3 | 5.4±0.3 | 41.1±4.3 | 12.3±0.3 | 1.91±0.15 | 0.12±0.01 |
| 65 | BH | FOUR | 10.31 | 19.70 | 10.03 | 1.50 | 0.75 | 0.62 | 1.01 | 2.99 | 0.100 | 0.132 | 0.030 | 1E-04 | 0.001 | 0.2239 | 1E-04 | 0.005 | 3.7±0.2 | 4.3±0.3 | 36.9±2.1 | 10.1±0.3 | 1.08±0.06 | 0.11±0.01 |
| 66 | BH | FOUR | 5.15 | 9.85 | 5.02 | 0.75 | 0.38 | 0.31 | 0.64 | 1.50 | 0.100 | 0.132 | 0.030 | 1E-04 | 0.001 | 0.2239 | 1E-04 | 0.005 | 2.4±0.2 | 4.0±0.2 | 22.8±2.9 | 7.7±0.4 | 0.56±0.07 | 0.07±0.01 |
| 67 | BH | FOUR | 2.58 | 4.93 | 2.51 | 0.37 | 0.19 | 0.16 | 0.45 | 0.75 | 0.100 | 0.132 | 0.030 | 1E-04 | 0.001 | 0.2239 | 1E-04 | 0.005 | 0.4±0.03 | 1.1±0.1 | 0.0±0.0 | 2.0±0.0 | 0.02±0.001 | 0.004±0.001 |
| 68 | BH | FOUR | 0.0 | 0.0 | 0.0 | 0.0 | 0.0 | 0.0 | 0.26 | 0.0 | 0.100 | 0.132 | 0.030 | 1E-04 | 0.001 | 0.2239 | 1E-04 | 0.005 | 0.5±0.03 | 1.1±0.05 | 0.0±0.0 | 2.0±0.0 | 0.03±0.001 | 0.002±0.001 |
| 69 | BH | FOUR | 20.61 | 39.41 | 20.04 | 2.99 | 1.50 | 1.25 | 1.68 | 5.99 | 0.050 | 0.066 | 0.015 | 5.0E-05 | 5.0E-04 | 0.2229 | 5.3E-05 | 0.0025 | 6.3±0.3 | 5.5±0.5 | 37.2±2.2 | 11.3±0.3 | 2.07±0.11 | 0.24±0.02 |
| 70 | BH | FOUR | 20.61 | 39.41 | 20.04 | 2.99 | 1.50 | 1.25 | 1.64 | 5.99 | 0.025 | 0.033 | 0.007 | 2.5E-05 | 2.5E-04 | 0.2224 | 2.6E-05 | 0.0012 | 4.8±0.4 | 4.5±0.2 | 14.9±4.1 | 10.8±0.4 | 1.85±0.23 | 0.33±0.02 |
| 71 | BH | FOUR | 20.61 | 39.41 | 20.04 | 2.99 | 1.50 | 1.25 | 1.62 | 5.99 | 0.013 | 0.016 | 0.004 | 1.3E-05 | 1.3E-04 | 0.2221 | 1.3E-05 | 6.0E-04 | 4.7±0.4 | 4.5±0.4 | 39.9±2.9 | 11.6±0.6 | 1.72±0.12 | 0.21±0.02 |
| 72 | BH | FOUR | 20.61 | 39.41 | 20.04 | 2.99 | 1.50 | 1.25 | 1.60 | 5.99 | 0.0 | 0.0 | 0.0 | 0.0 | 0.0 | 0.2219 | 0.0 | 0.0 | 2.0±0.5 | 0.9±0.2 | 0.0±0.0 | 7.0±1.3 | 0.59±0.24 | 0.01±0.001 |
| 73 | BT | ONE | 20.61 | 39.41 | 20.05 | 2.99 | 1.50 | 1.25 | 1.76 | 5.99 | 0.100 | 0.132 | 0.030 | 1E-04 | 0.001 | 0.2239 | 1E-04 | 0.005 | 2.9±0.2 | 3.9±0.3 | 20.0±2.1 | 16.3±0.9 | 0.62±0.06 | 0.07±0.01 |
| 74 | BT | ONE | 10.31 | 19.70 | 10.03 | 1.50 | 0.75 | 0.62 | 1.01 | 2.99 | 0.100 | 0.132 | 0.030 | 1E-04 | 0.001 | 0.2239 | 1E-04 | 0.005 | 3.0±0.1 | 2.7±0.2 | 22.7±2.2 | 17.2±0.7 | 0.57±0.04 | 0.08±0.01 |
| 75 | BT | ONE | 5.15 | 9.85 | 5.02 | 0.75 | 0.38 | 0.31 | 0.64 | 1.50 | 0.100 | 0.132 | 0.030 | 1E-04 | 0.001 | 0.2239 | 1E-04 | 0.005 | 1.5±0.2 | 1.7±0.1 | 5.9±1.8 | 10.0±1.3 | 0.23±0.04 | 0.04±0.01 |
| 76 | BT | ONE | 2.58 | 4.93 | 2.51 | 0.37 | 0.19 | 0.16 | 0.45 | 0.75 | 0.100 | 0.132 | 0.030 | 1E-04 | 0.001 | 0.2239 | 1E-04 | 0.005 | 1.1±0.2 | 1.7±0.1 | 1.5±0.6 | 7.5±0.9 | 0.18±0.03 | 0.02±0.003 |
| 77 | BT | ONE | 0.0 | 0.0 | 0.0 | 0.0 | 0.0 | 0.0 | 0.26 | 0.0 | 0.100 | 0.132 | 0.030 | 1E-04 | 0.001 | 0.2239 | 1E-04 | 0.005 | 0.3±0.02 | 1.2±0.1 | 0.0±0.0 | 2.0±0.0 | 0.02±0.001 | 0.002±0.001 |
| 78 | BT | ONE | 20.61 | 39.41 | 20.04 | 2.99 | 1.50 | 1.25 | 1.68 | 5.99 | 0.050 | 0.066 | 0.015 | 5.0E-05 | 5.0E-04 | 0.2229 | 5.3E-05 | 0.0025 | 2.2±0.4 | 2.3±0.4 | 0.0±0.0 | 8.4±2.2 | 0.41±0.14 | 0.07±0.02 |
| 79 | BT | ONE | 20.61 | 39.41 | 20.04 | 2.99 | 1.50 | 1.25 | 1.64 | 5.99 | 0.025 | 0.033 | 0.007 | 2.5E-05 | 2.5E-04 | 0.2224 | 2.6E-05 | 0.0012 | 5.5±0.5 | 3.0±0.3 | 3.6±2.3 | 19.2±1.4 | 0.98±0.13 | 0.05±0.01 |
| 80 | BT | ONE | 20.61 | 39.41 | 20.04 | 2.99 | 1.50 | 1.25 | 1.62 | 5.99 | 0.013 | 0.016 | 0.004 | 1.3E-05 | 1.3E-04 | 0.2221 | 1.3E-05 | 6.0E-04 | 6.8±0.3 | 3.7±0.3 | 4.4±1.6 | 22.0±0.8 | 1.51±0.08 | 0.14±0.01 |
| 81 | BT | ONE | 20.61 | 39.41 | 20.04 | 2.99 | 1.50 | 1.25 | 1.60 | 5.99 | 0.0 | 0.0 | 0.0 | 0.0 | 0.0 | 0.2219 | 0.0 | 0.0 | 5.1±0.4 | 2.2±0.3 | 0.0±0.0 | 17.2±1.4 | 0.95±0.12 | 0.04±0.02 |
| 82 | BT | TWO | 20.61 | 39.41 | 20.05 | 2.99 | 1.50 | 1.25 | 1.76 | 5.99 | 0.100 | 0.132 | 0.030 | 1E-04 | 0.001 | 0.2239 | 1E-04 | 0.005 | 2.2±0.2 | 4.2±0.2 | 17.8±2.9 | 13.6±0.6 | 0.45±0.05 | 0.06±0.008 |
| 83 | BT | TWO | 10.31 | 19.70 | 10.03 | 1.50 | 0.75 | 0.62 | 1.01 | 2.99 | 0.100 | 0.132 | 0.030 | 1E-04 | 0.001 | 0.2239 | 1E-04 | 0.005 | 0.7±0.2 | 1.5±0.2 | 2.2±1.6 | 4.8±1.0 | 0.09±0.03 | 0.02±0.006 |
| 84 | BT | TWO | 5.15 | 9.85 | 5.02 | 0.75 | 0.38 | 0.31 | 0.64 | 1.50 | 0.100 | 0.132 | 0.030 | 1E-04 | 0.001 | 0.2239 | 1E-04 | 0.005 | 0.9±0.2 | 1.3±0.1 | 4.6±1.6 | 6.3±1.2 | 0.17±0.04 | 0.03±0.007 |
| 85 | BT | TWO | 2.58 | 4.93 | 2.51 | 0.37 | 0.19 | 0.16 | 0.45 | 0.75 | 0.100 | 0.132 | 0.030 | 1E-04 | 0.001 | 0.2239 | 1E-04 | 0.005 | 0.4±0.01 | 1.0±0.04 | 0.0±0.0 | 2.0±0.0 | 0.03±0.002 | 0.003±0.001 |
| 86 | BT | TWO | 0.0 | 0.0 | 0.0 | 0.0 | 0.0 | 0.0 | 0.26 | 0.0 | 0.100 | 0.132 | 0.030 | 1E-04 | 0.001 | 0.2239 | 1E-04 | 0.005 | 0.3±0.01 | 1.3±0.2 | 0.0±0.0 | 2.0±0.0 | 0.03±0.001 | 0.002±0.001 |
| 87 | BT | TWO | 20.61 | 39.41 | 20.04 | 2.99 | 1.50 | 1.25 | 1.68 | 5.99 | 0.050 | 0.066 | 0.015 | 5.0E-05 | 5.0E-04 | 0.2229 | 5.3E-05 | 0.0025 | 1.8±0.4 | 2.5±0.3 | 0.0±0.0 | 8.8±1.5 | 0.29±0.10 | 0.02±0.009 |
| 88 | BT | TWO | 20.61 | 39.41 | 20.04 | 2.99 | 1.50 | 1.25 | 1.64 | 5.99 | 0.025 | 0.033 | 0.007 | 2.5E-05 | 2.5E-04 | 0.2224 | 2.6E-05 | 0.0012 | 5.8±0.3 | 5.8±0.3 | 37.4±1.3 | 21.9±1.0 | 1.32±0.08 | 0.11±0.01 |
| 89 | BT | TWO | 20.61 | 39.41 | 20.04 | 2.99 | 1.50 | 1.25 | 1.62 | 5.99 | 0.013 | 0.016 | 0.004 | 1.3E-05 | 1.3E-04 | 0.2221 | 1.3E-05 | 6.0E-04 | 5.4±0.2 | 5.9±0.3 | 38.9±2.6 | 22.1±0.8 | 1.05±0.07 | 0.11±0.01 |
| 90 | BT | TWO | 20.61 | 39.41 | 20.04 | 2.99 | 1.50 | 1.25 | 1.60 | 5.99 | 0.0 | 0.0 | 0.0 | 0.0 | 0.0 | 0.2219 | 0.0 | 0.0 | 5.4±0.7 | 2.3±0.3 | 0.0±0.0 | 17.2±1.4 | 0.86±0.15 | 0.02±0.004 |
| 91 | BT | THREE | 20.61 | 39.41 | 20.05 | 2.99 | 1.50 | 1.25 | 1.76 | 5.99 | 0.100 | 0.132 | 0.030 | 1E-04 | 0.001 | 0.2239 | 1E-04 | 0.005 | 3.1±0.2 | 4.6±0.3 | 34.0±1.9 | 15.8±0.5 | 0.72±0.05 | 0.08±0.01 |
| 92 | BT | THREE | 10.31 | 19.70 | 10.03 | 1.50 | 0.75 | 0.62 | 1.01 | 2.99 | 0.100 | 0.132 | 0.030 | 1E-04 | 0.001 | 0.2239 | 1E-04 | 0.005 | 1.1±0.2 | 2.3±0.4 | 3.3±1.6 | 5.7±1.2 | 0.18±0.05 | 0.05±0.02 |
| 93 | BT | THREE | 5.15 | 9.85 | 5.02 | 0.75 | 0.38 | 0.31 | 0.64 | 1.50 | 0.100 | 0.132 | 0.030 | 1E-04 | 0.001 | 0.2239 | 1E-04 | 0.005 | 0.4±0.1 | 0.9±0.2 | 0.6±0.5 | 2.9±0.6 | 0.05±0.02 | 0.02±0.005 |
| 94 | BT | THREE | 2.58 | 4.93 | 2.51 | 0.37 | 0.19 | 0.16 | 0.45 | 0.75 | 0.100 | 0.132 | 0.030 | 1E-04 | 0.001 | 0.2239 | 1E-04 | 0.005 | 0.2±0.01 | 0.6±0.03 | 0.0±0.0 | 2.0±0.0 | 0.01±0.001 | 0.003±0.001 |
| 95 | BT | THREE | 0.0 | 0.0 | 0.0 | 0.0 | 0.0 | 0.0 | 0.26 | 0.0 | 0.100 | 0.132 | 0.030 | 1E-04 | 0.001 | 0.2239 | 1E-04 | 0.005 | 0.3±0.02 | 1.6±0.1 | 0.0±0.0 | 2.0±0.0 | 0.03±0.002 | 0.002±0.001 |
| 96 | BT | THREE | 20.61 | 39.41 | 20.04 | 2.99 | 1.50 | 1.25 | 1.68 | 5.99 | 0.050 | 0.066 | 0.015 | 5.0E-05 | 5.0E-04 | 0.2229 | 5.3E-05 | 0.0025 | 1.7±0.4 | 1.9±0.4 | 0.0±0.0 | 7.6±1.3 | 0.25±0.09 | 0.03±0.02 |
| 97 | BT | THREE | 20.61 | 39.41 | 20.04 | 2.99 | 1.50 | 1.25 | 1.64 | 5.99 | 0.025 | 0.033 | 0.007 | 2.5E-05 | 2.5E-04 | 0.2224 | 2.6E-05 | 0.0012 | 4.6±0.1 | 5.1±0.3 | 31.0±2.2 | 19.8±0.9 | 0.87±0.04 | 0.05±0.01 |
| 98 | BT | THREE | 20.61 | 39.41 | 20.04 | 2.99 | 1.50 | 1.25 | 1.62 | 5.99 | 0.013 | 0.016 | 0.004 | 1.3E-05 | 1.3E-04 | 0.2221 | 1.3E-05 | 6.0E-04 | 5.4±0.3 | 5.4±0.2 | 35.1±2.7 | 20.4±0.6 | 1.00±0.03 | 0.07±0.01 |
| 99 | BT | THREE | 20.61 | 39.41 | 20.04 | 2.99 | 1.50 | 1.25 | 1.60 | 5.99 | 0.0 | 0.0 | 0.0 | 0.0 | 0.0 | 0.2219 | 0.0 | 0.0 | 5.7±0.6 | 2.4±0.3 | 0.0±0.0 | 18.8±0.8 | 1.01±0.09 | 0.02±0.003 |
| 100 | BT | FOUR | 20.61 | 39.41 | 20.05 | 2.99 | 1.50 | 1.25 | 1.76 | 5.99 | 0.100 | 0.132 | 0.030 | 1E-04 | 0.001 | 0.2239 | 1E-04 | 0.005 | 4.7±0.3 | 5.0±0.2 | 27.6±2.0 | 18.2±1.1 | 1.05±0.09 | 0.09±0.01 |
| 101 | BT | FOUR | 10.31 | 19.70 | 10.03 | 1.50 | 0.75 | 0.62 | 1.01 | 2.99 | 0.100 | 0.132 | 0.030 | 1E-04 | 0.001 | 0.2239 | 1E-04 | 0.005 | 2.8±0.1 | 3.3±0.2 | 30.3±1.4 | 15.4±0.4 | 0.47±0.02 | 0.08±0.01 |
| 102 | BT | FOUR | 5.15 | 9.85 | 5.02 | 0.75 | 0.38 | 0.31 | 0.64 | 1.50 | 0.100 | 0.132 | 0.030 | 1E-04 | 0.001 | 0.2239 | 1E-04 | 0.005 | 2.4±0.4 | 3.4±0.6 | 18.6±3.4 | 12.2±1.8 | 0.37±0.07 | 0.09±0.02 |
| 103 | BT | FOUR | 2.58 | 4.93 | 2.51 | 0.37 | 0.19 | 0.16 | 0.45 | 0.75 | 0.100 | 0.132 | 0.030 | 1E-04 | 0.001 | 0.2239 | 1E-04 | 0.005 | 0.4±0.03 | 0.9±0.04 | 0.0±0.0 | 2.3±0.1 | 0.03±0.003 | 0.03±0.02 |
| 104 | BT | FOUR | 0.0 | 0.0 | 0.0 | 0.0 | 0.0 | 0.0 | 0.26 | 0.0 | 0.100 | 0.132 | 0.030 | 1E-04 | 0.001 | 0.2239 | 1E-04 | 0.005 | 0.3±0.01 | 2.0±0.1 | 0.0±0.0 | 2.0±0.0 | 0.03±0.002 | 0.003±0.001 |
| 105 | BT | FOUR | 20.61 | 39.41 | 20.04 | 2.99 | 1.50 | 1.25 | 1.68 | 5.99 | 0.050 | 0.066 | 0.015 | 5.0E-05 | 5.0E-04 | 0.2229 | 5.3E-05 | 0.0025 | 1.9±0.3 | 2.5±0.4 | 0.0±0.0 | 6.8±0.8 | 0.30±0.10 | 0.04±0.02 |
| 106 | BT | FOUR | 20.61 | 39.41 | 20.04 | 2.99 | 1.50 | 1.25 | 1.64 | 5.99 | 0.025 | 0.033 | 0.007 | 2.5E-05 | 2.5E-04 | 0.2224 | 2.6E-05 | 0.0012 | 4.4±0.2 | 5.5±0.4 | 33.6±2.9 | 19.8±0.4 | 0.91±0.02 | 0.07±0.01 |
| 107 | BT | FOUR | 20.61 | 39.41 | 20.04 | 2.99 | 1.50 | 1.25 | 1.62 | 5.99 | 0.013 | 0.016 | 0.004 | 1.3E-05 | 1.3E-04 | 0.2221 | 1.3E-05 | 6.0E-04 | 5.1±0.2 | 4.9±0.3 | 35.3±1.9 | 20.7±0.6 | 0.90±0.04 | 0.07±0.01 |
| 108 | BT | FOUR | 20.61 | 39.41 | 20.04 | 2.99 | 1.50 | 1.25 | 1.60 | 5.99 | 0.0 | 0.0 | 0.0 | 0.0 | 0.0 | 0.2219 | 0.0 | 0.0 | 5.8±0.3 | 2.0±0.2 | 0.0±0.0 | 18.4±0.4 | 0.91±0.06 | 0.03±0.004 |

Table S2. ANOVA summary tables for each parameter analyzed (SL, RL, PN, LN, AFW, RFW), according to the three factors employed: Genotype (Gen.), Number of Subcultures (Subc.) and Medium and their interactions. SS: sum of squares, df: Degrees of Freedom, MS: Mean Squares, F: statistical F value, p: probability. Asterisks indicate significant effect (α=0.01).

| **SL** | **SS** | **df** | **MS** | **F** | **p** |
| --- | --- | --- | --- | --- | --- |
| Intercept | 4096.83 | 1 | 4096.83 | 4548.90 | <0.001* |
| Gen | 404.91 | 2 | 202.46 | 224.80 | <0.001* |
| Subc | 41.04 | 3 | 13.68 | 15.19 | <0.001* |
| Medium | 1412.67 | 8 | 176.58 | 196.07 | <0.001* |
| Gen*Subc | 17.18 | 6 | 2.86 | 3.18 | 0.004 |
| Gen*Medium | 678.80 | 16 | 42.43 | 47.11 | <0.001* |
| Subc*Medium | 174.94 | 24 | 7.29 | 8.09 | <0.001* |
| Gen*Subc*Medium | 178.57 | 48 | 3.72 | 4.13 | <0.001* |
| Error | 1041.12 | 1156 | 0.90 |  |  |

| **RL** | **SS** | **df** | **MS** | **F** | **p** |
| --- | --- | --- | --- | --- | --- |
| Intercept | 5427.57 | 1 | 5427.57 | 3493.81 | <0.001* |
| Gen | 13.23 | 2 | 6.61 | 4.26 | 0.014 |
| Subc | 138.70 | 3 | 46.24 | 29.76 | <0.001* |
| Medium | 917.55 | 8 | 114.69 | 73.83 | <0.001* |
| Gen*Subc | 19.04 | 6 | 3.17 | 2.04 | 0.057 |
| Gen*Medium | 210.51 | 16 | 13.16 | 8.47 | <0.001* |
| Subc*Medium | 480.22 | 24 | 20.01 | 12.88 | <0.001* |
| Gen*Subc*Medium | 360.06 | 48 | 7.50 | 4.83 | <0.001* |
| Error | 1795.82 | 1156 | 1.55 |  |  |

| **PN** | **SS** | **df** | **MS** | **F** | **p** |
| --- | --- | --- | --- | --- | --- |
| Intercept | 81042.2 | 1 | 81042.24 | 1159.34 | <0.001* |
| Gen | 7335.3 | 2 | 3667.63 | 52.47 | <0.001* |
| Subc | 10948.7 | 3 | 3649.55 | 52.21 | <0.001* |
| Medium | 37790.2 | 8 | 4723.77 | 67.58 | <0.001* |
| Gen*Subc | 932.3 | 6 | 155.39 | 2.22 | 0.039 |
| Gen*Medium | 20257.5 | 16 | 1266.09 | 18.11 | <0.001* |
| Subc*Medium | 38607.6 | 24 | 1608.65 | 23.01 | <0.001* |
| Gen*Subc*Medium | 34253.0 | 48 | 713.60 | 10.21 | <0.001* |
| Error | 80808.7 | 1156 | 69.90 |  |  |

| **LN** | **SS** | **df** | **MS** | **F** | **p** |
| --- | --- | --- | --- | --- | --- |
| Intercept | 48610.51 | 1 | 48610.51 | 6698.02 | <0.001* |
| Gen | 5776.43 | 2 | 2888.22 | 397.97 | <0.001* |
| Subc | 400.95 | 3 | 133.65 | 18.42 | <0.001* |
| Medium | 9139.23 | 8 | 1142.40 | 157.41 | <0.001* |
| Gen*Subc | 193.03 | 6 | 32.17 | 4.43 | <0.001* |
| Gen*Medium | 4454.38 | 16 | 278.40 | 38.36 | <0.001* |
| Subc*Medium | 1891.35 | 24 | 78.81 | 10.86 | <0.001* |
| Gen*Subc*Medium | 1186.93 | 48 | 24.73 | 3.41 | <0.001* |
| Error | 8389.61 | 1156 | 7.26 |  |  |

| **AFW** | **SS** | **df** | **MS** | **F** | **p** |
| --- | --- | --- | --- | --- | --- |
| Intercept | 270.61 | 1 | 270.61 | 2204.91 | <0.001* |
| Gen | 53.53 | 2 | 26.77 | 218.10 | <0.001* |
| Subc | 3.67 | 3 | 1.22 | 9.96 | <0.001* |
| Medium | 145.30 | 8 | 18.16 | 147.98 | <0.001* |
| Gen*Subc | 1.60 | 6 | 0.27 | 2.18 | 0.043 |
| Gen*Medium | 71.37 | 16 | 4.46 | 36.35 | <0.001* |
| Subc*Medium | 20.91 | 24 | 0.87 | 7.10 | <0.001* |
| Gen*Subc*Medium | 23.31 | 48 | 0.49 | 3.96 | <0.001* |
| Error | 141.88 | 1156 | 0.12 |  |  |

| **RFW** | **SS** | **df** | **MS** | **F** | **p** |
| --- | --- | --- | --- | --- | --- |
| Intercept | 2.98 | 1 | 2.98 | 1141.01 | <0.001* |
| Gen | 0.89 | 2 | 0.44 | 170.51 | <0.001* |
| Subc | 0.06 | 3 | 0.02 | 7.08 | <0.001* |
| Medium | 1.38 | 8 | 0.17 | 65.97 | <0.001* |
| Gen*Subc | 0.02 | 6 | 0.004 | 1.35 | 0.231 |
| Gen*Medium | 0.98 | 16 | 0.06 | 23.45 | <0.001* |
| Subc*Medium | 0.47 | 24 | 0.02 | 7.55 | <0.001* |
| Gen*Subc*Medium | 0.83 | 48 | 0.02 | 6.60 | <0.001* |
| Error | 3.02 | 1156 | 0.003 |  |  |

Table S3. ‘IF-THEN’ rules generated by ANN modelling. Inputs with the highest membership degree, showing the major contibutions for each response on every output, are indicated in bold. Genot: genotype; Subcult: number of subcultures.

| **Rules** |  | **Genot** | **Subcult** | **NH_4_^+^** | **SO_4_^2-^** | **Na^+^** | **Cu^2+^** | **MoO_4_^2-^** |  | **SL** | **RL** | **PN** | **LN** | **AFW** | **RFW** | **Membership** |
| --- | --- | --- | --- | --- | --- | --- | --- | --- | --- | --- | --- | --- | --- | --- | --- | --- |
| 1 | I  F |  |  | Low |  |  |  |  | T  H  E  N | Low |  |  |  |  |  | 0.98 |
| 2 |  |  |  | High |  |  |  |  |  | High |  |  |  |  |  | 0.83 |
| 3 |  | BH |  |  |  |  | Low |  |  | Low |  |  |  |  |  | 0.76 |
| 4 |  | BH |  |  |  |  | High |  |  | Low |  |  |  |  |  | 0.71 |
| **5** |  | **BD** |  |  |  |  | **Low** |  |  | **Low** |  |  |  |  |  | **1.00** |
| 6 |  | BD |  |  |  |  | High |  |  | Low |  |  |  |  |  | 1.00 |
| **7** |  | **BT** |  |  |  |  | **Low** |  |  | **High** |  |  |  |  |  | **0.77** |
| 8 |  | BT |  |  |  |  | High |  |  | Low |  |  |  |  |  | 1.00 |
| **9** | I  F | **BH** |  |  | **Low** |  |  | **Low** | T  H  E  N |  | **Low** |  |  |  |  | **1.00** |
| 10 |  | BD |  |  | Low |  |  | Low |  |  | Low |  |  |  |  | 1.00 |
| 11 |  | BT |  |  | Low |  |  | Low |  |  | Low |  |  |  |  | 1.00 |
| 12 |  | BH |  |  | Low |  |  | Mid |  |  | High |  |  |  |  | 1.00 |
| 13 |  | BD |  |  | Low |  |  | Mid |  |  | High |  |  |  |  | 1.00 |
| **14** |  | **BT** |  |  | **Low** |  |  | **Mid** |  |  | **High** |  |  |  |  | **1.00** |
| 15 |  | BH |  |  | Low |  |  | High |  |  | Low |  |  |  |  | 1.00 |
| 16 |  | BD |  |  | Low |  |  | High |  |  | Low |  |  |  |  | 1.00 |
| 17 |  | BT |  |  | Low |  |  | High |  |  | Low |  |  |  |  | 1.00 |
| 18 |  | BH |  |  | High |  |  | Low |  |  | High |  |  |  |  | 1.00 |
| 19 |  | BD |  |  | High |  |  | Low |  |  | High |  |  |  |  | 1.00 |
| 20 |  | BT |  |  | High |  |  | Low |  |  | High |  |  |  |  | 1.00 |
| 21 |  | BH |  |  | High |  |  | Mid |  |  | High |  |  |  |  | 1.00 |
| 22 |  | BD |  |  | High |  |  | Mid |  |  | High |  |  |  |  | 1.00 |
| 23 |  | BT |  |  | High |  |  | Mid |  |  | Low |  |  |  |  | 1.00 |
| 24 |  | BH |  |  | High |  |  | High |  |  | Low |  |  |  |  | 1.00 |
| 25 |  | BD |  |  | High |  |  | High |  |  | Low |  |  |  |  | 1.00 |
| 26 |  | BT |  |  | High |  |  | High |  |  | Low |  |  |  |  | 1.00 |
| 27 |  |  | ONE |  |  | Low |  |  |  |  | Low |  |  |  |  | 1.00 |
| 28 |  |  | ONE |  |  | High |  |  |  |  | High |  |  |  |  | 1.00 |
| 29 |  |  | TWO |  |  | Low |  |  |  |  | Low |  |  |  |  | 1.00 |
| 30 |  |  | TWO |  |  | High |  |  |  |  | High |  |  |  |  | 1.00 |
| 31 |  |  | THREE |  |  | Low |  |  |  |  | Low |  |  |  |  | 1.00 |
| 32 |  |  | THREE |  |  | High |  |  |  |  | High |  |  |  |  | 1.00 |
| 33 |  |  | FOUR |  |  | Low |  |  |  |  | Low |  |  |  |  | 1.00 |
| 34 |  |  | FOUR |  |  | High |  |  |  |  | High |  |  |  |  | 1.00 |
| **35** | I  F | **BH** |  |  | **Low** |  |  | **Low** | T  H  E  N |  |  | **Low** |  |  |  | **1.00** |
| 36 |  | BD |  |  | Low |  |  | Low |  |  |  | Low |  |  |  | 1.00 |
| 37 |  | BT |  |  | Low |  |  | Low |  |  |  | Low |  |  |  | 1.00 |
| 38 |  | BH |  |  | Low |  |  | Mid |  |  |  | Low |  |  |  | 1.00 |
| 39 |  | BD |  |  | Low |  |  | Mid |  |  |  | High |  |  |  | 1.00 |
| **40** |  | **BT** |  |  | **Low** |  |  | **Mid** |  |  |  | **High** |  |  |  | **1.00** |
| 41 |  | BH |  |  | Low |  |  | High |  |  |  | Low |  |  |  | 1.00 |
| 42 |  | BD |  |  | Low |  |  | High |  |  |  | Low |  |  |  | 1.00 |
| 43 |  | BT |  |  | Low |  |  | High |  |  |  | Low |  |  |  | 1.00 |
| 44 |  | BH |  |  | High |  |  | Low |  |  |  | High |  |  |  | 1.00 |
| 45 |  | BD |  |  | High |  |  | Low |  |  |  | High |  |  |  | 1.00 |
| 46 |  | BT |  |  | High |  |  | Low |  |  |  | High |  |  |  | 1.00 |
| 47 |  | BH |  |  | High |  |  | Mid |  |  |  | High |  |  |  | 1.00 |
| 48 |  | BD |  |  | High |  |  | Mid |  |  |  | Low |  |  |  | 1.00 |
| 49 |  | BT |  |  | High |  |  | Mid |  |  |  | Low |  |  |  | 1.00 |
| 50 |  | BH |  |  | High |  |  | High |  |  |  | Low |  |  |  | 1.00 |
| 51 |  | BD |  |  | High |  |  | High |  |  |  | Low |  |  |  | 1.00 |
| 52 |  | BT |  |  | High |  |  | High |  |  |  | Low |  |  |  | 1.00 |
| 53 |  |  | ONE |  |  | Low |  |  |  |  |  | Low |  |  |  | 1.00 |
| 54 |  |  | ONE |  |  | High |  |  |  |  |  | High |  |  |  | 1.00 |
| 55 |  |  | TWO |  |  | Low |  |  |  |  |  | Low |  |  |  | 1.00 |
| 56 |  |  | TWO |  |  | High |  |  |  |  |  | High |  |  |  | 1.00 |
| 57 |  |  | THREE |  |  | Low |  |  |  |  |  | Low |  |  |  | 1.00 |
| 58 |  |  | THREE |  |  | High |  |  |  |  |  | High |  |  |  | 1.00 |
| 59 |  |  | FOUR |  |  | Low |  |  |  |  |  | Low |  |  |  | 1.00 |
| 60 |  |  | FOUR |  |  | High |  |  |  |  |  | High |  |  |  | 1.00 |
| 61 | I  F | BH |  | Low |  |  |  |  | T  H  E  N |  |  |  | Low |  |  | 0.95 |
| 62 |  | BH |  | High |  |  |  |  |  |  |  |  | Low |  |  | 0.59 |
| **63** |  | **BD** |  | **Low** |  |  |  |  |  |  |  |  | **Low** |  |  | **0.96** |
| 64 |  | BD |  | High |  |  |  |  |  |  |  |  | Low |  |  | 0.80 |
| 65 |  | BT |  | Low |  |  |  |  |  |  |  |  | Low |  |  | 0.96 |
| **66** |  | **BT** |  | **High** |  |  |  |  |  |  |  |  | **High** |  |  | **0.74** |
| **67** | I  F | **BH** |  |  | **Low** |  |  | **Low** | T  H  E  N |  |  |  |  | **Low** |  | **1.00** |
| 68 |  | BD |  |  | Low |  |  | Low |  |  |  |  |  | Low |  | 1.00 |
| 69 |  | BT |  |  | Low |  |  | Low |  |  |  |  |  | Low |  | 1.00 |
| 70 |  | BH |  |  | Low |  |  | Mid |  |  |  |  |  | Low |  | 1.00 |
| 71 |  | BD |  |  | Low |  |  | Mid |  |  |  |  |  | Low |  | 1.00 |
| 72 |  | **BT** |  |  | **Low** |  |  | **Mid** |  |  |  |  |  | **High** |  | **1.00** |
| 73 |  | BH |  |  | Low |  |  | High |  |  |  |  |  | Low |  | 1.00 |
| 74 |  | BD |  |  | Low |  |  | High |  |  |  |  |  | Low |  | 0.98 |
| 75 |  | BT |  |  | Low |  |  | High |  |  |  |  |  | Low |  | 1.00 |
| 76 |  | BH |  |  | High |  |  | Low |  |  |  |  |  | High |  | 1.00 |
| 77 |  | BD |  |  | High |  |  | Low |  |  |  |  |  | High |  | 1.00 |
| 78 |  | BT |  |  | High |  |  | Low |  |  |  |  |  | High |  | 1.00 |
| 79 |  | BH |  |  | High |  |  | Mid |  |  |  |  |  | High |  | 1.00 |
| 80 |  | BD |  |  | High |  |  | Mid |  |  |  |  |  | High |  | 1.00 |
| 81 |  | BT |  |  | High |  |  | Mid |  |  |  |  |  | Low |  | 1.00 |
| 82 |  | BH |  |  | High |  |  | High |  |  |  |  |  | High |  | 0.71 |
| 83 |  | BD |  |  | High |  |  | High |  |  |  |  |  | Low |  | 0.76 |
| 84 |  | BT |  |  | High |  |  | High |  |  |  |  |  | Low |  | 0.70 |
| **85** |  | **BH** |  |  | **Low** |  | **Low** |  |  |  |  |  |  |  | **Low** | **1.00** |
| 86 |  | BH |  |  | Low |  | High |  |  |  |  |  |  |  | High | 1.00 |
| **87** |  | **BH** |  |  | **High** |  | **Low** |  |  |  |  |  |  |  | **High** | **1.00** |
| 88 |  | BH |  |  | High |  | High |  |  |  |  |  |  |  | High | 1.00 |
| 89 |  | BD |  |  | Low |  | Low |  |  |  |  |  |  |  | Low | 1.00 |
| 90 |  | BD |  |  | Low |  | High |  |  |  |  |  |  |  | High | 1.00 |
| 91 |  | BD |  |  | High |  | Low |  |  |  |  |  |  |  | High | 1.00 |
| 92 |  | BD |  |  | High |  | High |  |  |  |  |  |  |  | High | 1.00 |
| 93 |  | BT |  |  | Low |  | Low |  |  |  |  |  |  |  | Low | 1.00 |
| 94 |  | BT |  |  | Low |  | High |  |  |  |  |  |  |  | High | 1.00 |
| 95 |  | BT |  |  | High |  | Low |  |  |  |  |  |  |  | High | 1.00 |
| 96 |  | BT |  |  | High |  | High |  |  |  |  |  |  |  | High | 1.00 |
| 97 |  |  |  |  |  |  |  | Low |  |  |  |  |  |  | Low | 1.00 |
| 98 |  |  |  |  |  |  |  | Mid |  |  |  |  |  |  | Low | 1.00 |
| 99 |  |  |  |  |  |  |  | High |  |  |  |  |  |  | Low | 1.00 |


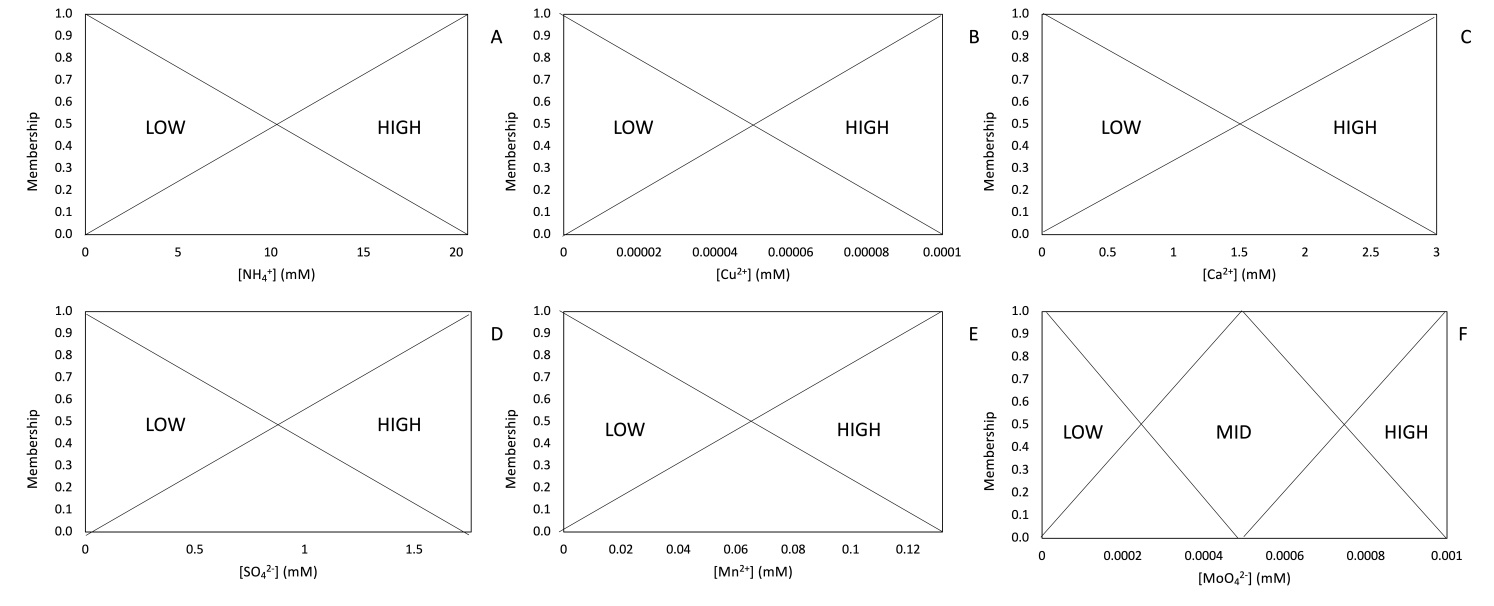


Figure S1. Graphical ranging of each ion concentrations offered by ANN model for each output. (A) NH_4_^+^ concentrations for SL and LN. (B) SO_4_^2-^ concentrations for RL, PN, AFW and RFW. (C) Cu^2+^ concentrations for SL and RFW. (D) MoO_4_^2-^ concentrations for RL, PN, AFW and RFW. (E) Na^+^ concentrations for RL and PN.
